# Variation in worldwide incidence of Guillain-Barré syndrome: a population-based study in urban China and existing global evidence

**DOI:** 10.3389/fimmu.2024.1415986

**Published:** 2024-09-10

**Authors:** Lu Xu, Chen Zhao, Yutong Bao, Yuchen Liu, Yuqing Liang, Jiyu Wei, Guozhen Liu, Jinxi Wang, Siyan Zhan, Shengfeng Wang, Dongsheng Fan

**Affiliations:** ^1^ Research Center of Clinical Epidemiology, Peking University Third Hospital, Beijing, China; ^2^ Key Laboratory of Epidemiology of Major Diseases, Peking University, Ministry of Education, Beijing, China; ^3^ Department of Neurology, Peking University Third Hospital, Beijing, China; ^4^ Department of Research & Development, Peking University Health Information Technology Co., Ltd., Beijing, China; ^5^ Department of Strategic Planning, Shanghai Songsheng Business Consulting Co., Ltd., Beijing, China; ^6^ Department of Epidemiology and Biostatistics, School of Public Health, Peking University, Beijing, China; ^7^ Center for Intelligent Public Health, Institute for Artificial Intelligence, Peking University, Beijing, China; ^8^ Key Laboratory for Neuroscience, National Health Commission/Ministry of Education, Peking University, Beijing, China; ^9^ Beijing Key Laboratory of Biomarker and Translational Research in Neurodegenerative Diseases, Beijing, China

**Keywords:** Guillain-Barré syndrome, incidence, epidemiology, population-based, global evidence

## Abstract

**Background and objectives:**

Geographical variation existed in the incidences of Guillain-Barré syndrome (GBS), but no national population-based study has evaluated the incidences of GBS in China. This study aimed to estimate the incidence of GBS in urban China and evaluate the worldwide variation in the incidence of GBS.

**Methods:**

Firstly, we did a population-based study to calculate the incidence of GBS in urban China based on the National Urban Medical Insurance database from 2013 to 2017. To identify GBS cases, natural language processing was used first for handling the lengthy and unstructured diagnostic information and then checked by prestigious neurologists. Secondly, a systematic review and meta-analysis were performed to analyze the incidence of GBS worldwide. Up to July 4, 2022, Medline, Embase, and Web of Science were retrieved to identify the population-based studies regarding the incidence of GBS. The basic information and the statistics regarding incidence were extracted. Quality assessment considered sample representativeness, condition assessment, and statistical methods.

**Results:**

A total of 1.44 billion person-years in insurance data was covered, with 3,534 GBS cases identified. The annual incidences of GBS in urban China between 2013 and 2017 ranged from 0.41 (95% CI: 0.27 to 0.58) to 0.58 (95% CI: 0.38 to 0.82) per 100,000 person-years. The incidence was the highest in Northwest China and the lowest in Northeast China. The meta-analysis included 122 articles. The quality assessment showed that the quality scores of 43.3% of studies were ≥ 0.75 (the total score is 1). The global incidence of GBS was 1.12 (95% CI: 0.98 to 1.27) per 100,000 person-years. The incidences in West Europe, South Asia, and North Europe were higher, while the incidences in Australia and New Zealand, Southeast Asia, and North Africa were lower. The incidence of enteric infections was positively associated with the incidence of GBS (coefficient=0.0000185, P=0.007). The incidence in Europe, Australia, and America rose significantly from 1960 to 2020 (coefficient=0.01, t=2.52, P=0.015).

**Discussion:**

There is a clear regional variation of the GBS incidence at both national and global levels. Careful control of enteric infections should be conducted to reduce the disease burden.

## 1 Introduction

Guillain-Barré syndrome (GBS) is an immune-mediated acute polyradiculoneuropathy which is the most common cause of acute flaccid paralysis across the world (1). Although GBS is classically considered a demyelinating neuropathy caused by macrophage-induced phagocytosis of myelin (2), the axonal form has now been widely recognized as another major subtype of GBS (3). Approximately 1 in 5 patients cannot walk independently at 1 year from disease onset (1) and the years lived with disability were 44,407 in 2019 (4). Even when treated with standard immunotherapies the mortality of GBS remains high and 3%-7% of patients die (5, 6). The annual median cost for treating GBS was estimated at 16,428 American dollars (USD) in South Korea (7), and the estimated cost per patient was 318,966 USD in the US (8). Also, the Global Burden of Diseases Study (GBD) found a 6.4% increase in the prevalence of GBS between 1990 and 2019 (4). Therefore, the disease burden of GBS is heavy and gradually increasing.

Incidences of GBS have been reported in many countries ranging from 0.38 to 2.53 per 100,000 person-years (9). Previous studies suggested a geographical variation in annual incidences of GBS. Most studies investigated populations in Europe and North America reporting annual incidences between 0.84 and 1.91 per 100,000 person-years (9), whereas incidences of Asian populations were much lower with 0.44 per 100,000 person-years in Japan (10) and 0.63 per 100,000 person-years in 2018 in Korea (11). Studying the regional variation of GBS incidence and the underlying reasons can provide a better understanding of the disease and potential methods to reduce the disease burden. By far, there is only one hospital-based study which incorporated all tertiary hospitals in China reporting an incidence of GBS as 0.698 per 100,000 person-years (12). However, hospital-based studies can lead to an inevitable selection bias and it is hard to identify the source population of the patients. Population-based studies can provide robust estimates, which are urgently needed in China.

We performed a nationwide population-based investigation of the incidence of GBS in urban China using the Urban Employee Basic Medical Insurance (UEBMI) database and the Urban Residence Basic Medical Insurance (URBMI) database. Moreover, to compare our national result with the global GBS burden and describe the patterns of GBS incidence worldwide, we conducted a systematic review and meta-analysis of published studies reporting GBS incidence which has not been updated over 10 years (13).

## 2 Methods

### 2.1 Population-based study in urban China

#### 2.1.1 Study population

The National Urban Medical Insurance database which has covered over 95% of the whole urban Chinese population was used in this study, including the UEBMI database for working and retired employees in cities and the URBMI database for urban citizens without employment (14). The information in the UEBMI and URBMI databases included the sociodemographic characteristics, medical treatment records, and medical expenditure of insured people.

To calculate the incidence of GBS in urban China, all urban insured people in the UEBMI and URBMI databases of 23 provinces from 2013 to 2017 were used as the study population in this study. Of the 31 provinces in mainland China, 8 provinces were excluded due to lack of diagnostic information, such as the International Classification of Diseases (ICD) code or diagnostic text; reporting policy exemptions; only available to UEBMI or URBMI database; or absence or abnormality of other important information, consistent with previously published studies (15–17). The comparison of the 8 excluded provinces and the 23 included provinces can be seen in **Supplementary Data Sheet 1**.

#### 2.1.2 Identification of patients with GBS

Since the reimbursement records will be recorded in the UEBMI or URBMI database if the national insurance card is provided for the medical service (even though no medical expenditure is reimbursed), UEBMI or URBMI database can capture all patients with GBS in urban China. To identify the patients with GBS, firstly, natural language processing was used to handle the lengthy and unstructured diagnostic information in the UEBMI or URBMI database to identify the patients with GBS according to the medical terms and the ICD-10 codes regarding “Guillain-Barré syndrome”, “acute inflammatory demyelinating polyradiculoneuropathy”, “acute motor axonal neuropathy”, “acute motor sensory axonal neuropathy”, and “Miller-Fisher syndrome”. The detailed medical terms and ICD-10 codes used are shown in **Supplementary Data Sheet 2**. The potential GBS cases identified by the natural language processing were then checked by two prestigious neurologists independently, with discrepancies between them solved by consulting with another senior neurologist.

#### 2.1.3 Statistical analysis

The annual incidence of GBS in urban China was calculated by a two-stage approach, which was also used in our previous studies (15, 18–21). The detailed calculation approach can be seen in **Supplementary Data Sheet 3**. Age-adjusted incidences were calculated based on the 2010 Chinese national census data. Subgroup analyses were performed by sex, age, and geographical areas (East, North, Northeast, Northwest, Southcentral, and Southwest). Since diagnostic information in part of the claim records was missing, two sensitivity analyses were performed: 1) only considering the target patients from the individuals not missing diagnostic information (estimating the lower bound of the incidence); 2) excluding the top 10% of the provinces ranked by the missing diagnosis rate. All statistical analyses were conducted using Stata 15.0 (StataCorp, College Station, TX, USA).

### 2.2 Systematic review and meta-analysis

#### 2.2.1 Inclusion and exclusion criteria for literatures

Databases including Medline, Embase, and Web of Science were retrieved to identify relevant articles published up to July 4, 2022. The search strategy can be found in **Supplementary Data Sheet 4**. Also, we screened the references of all relevant studies to identify additional eligible articles. The inclusion criterion was population-based studies reporting the incidence of GBS or providing the data to compute the incidence of GBS. The result of the GBS incidence in urban China in this study was also included. Exclusion criteria were: 1) duplicates; 2) reviews, systematic reviews, meta-analyses, and books; 3) biomarkers, neuropathology, and experiments; 4) clinical trials; 5) case reports, case study, case series study, and case-control study; 6) Epidemiological studies with other aims; 7) not in English; 8) no full text. Two reviewers assessed all articles independently, with disagreements between them solved by consulting with another senior reviewer. This study was registered in the PROSPERO, number CRD42024524382. The review protocol can be accessed in https://www.crd.york.ac.uk/PROSPERO/.

#### 2.2.2 Data extraction and quality evaluation

The data extraction form contained the basic information of the articles and the statistics regarding incidence. The quality assessment tool applied (**Supplementary Data Sheet 5**) (22, 23) covered three aspects: sample representativeness, condition assessment, and statistical methods. Data extraction and quality evaluation were done by two reviewers independently, with disagreements between them solved by consulting with another senior reviewer.

#### 2.2.3 Evidence synthesis

Pooled incidence was synthesized by using the incidence values and their corresponding standard errors provided by included studies or estimated from the other provided statistics. The Freeman-Tukey double arcsine transformation was used to stabilize the variance of study-specific incidence.

Subgroup analyses were done by sex, age, subcontinent (https://unstats.un.org/unsd/methodology/m49/, retrieved October 5, 2022), and season. If a subgroup-specific incidence during multiple periods was reported in a study, the most recent one was considered. To evaluate the impacts of the socio-demographic index (SDI), physician density (per 10,000 population), human development index (HDI), proportion of elderly population, incidence of enteric infections (per 100,000 person-years), and incidence of respiratory infections (per 100,000 person-years) on GBS incidence, linear regression was used to evaluate the association between them and national incidences. Data on SDI, physician density, enteric infections, and respiratory infections were obtained from GBD 2019. Data on HDI were obtained from the report of the United Nations Development Programme. Data on the proportion of the elderly population were obtained from the Organization for Economic Co-operation and Development (OECD). The temporal trend in incidence was evaluated by fitting a linear regression of the arcsine transformation of annual incidence and the year. If the period for an incidence was longer than one year but no longer than 10 years, the incidence represented the median year (24). *P*-value < 0.05 in the linear regression was considered statistically significant. *I^2^* was used to assess the heterogeneity between studies, with *I^2^* values greater than 60–70% indicating substantial heterogeneity (25). Meta-regression was done to evaluate the sources of heterogeneity by including subcontinent, study period, study quality, economic level, average age, and male-to-female ratio. Publication bias was detected by the Egger’s test and funnel plot. The meta-analysis was done by the metan package in Stata 15.0 (StataCorp, College Station, TX, USA).

### 2.3 Standard protocol approvals, registrations, and patient consents

This study was approved by the ethical review committee of the Peking University Health Science Center (Institutional Review Board No.: IRB00001052-18012), and informed consent was waived.

## 3 Results

### 3.1 Population-based study in urban China

**Figure 1:**
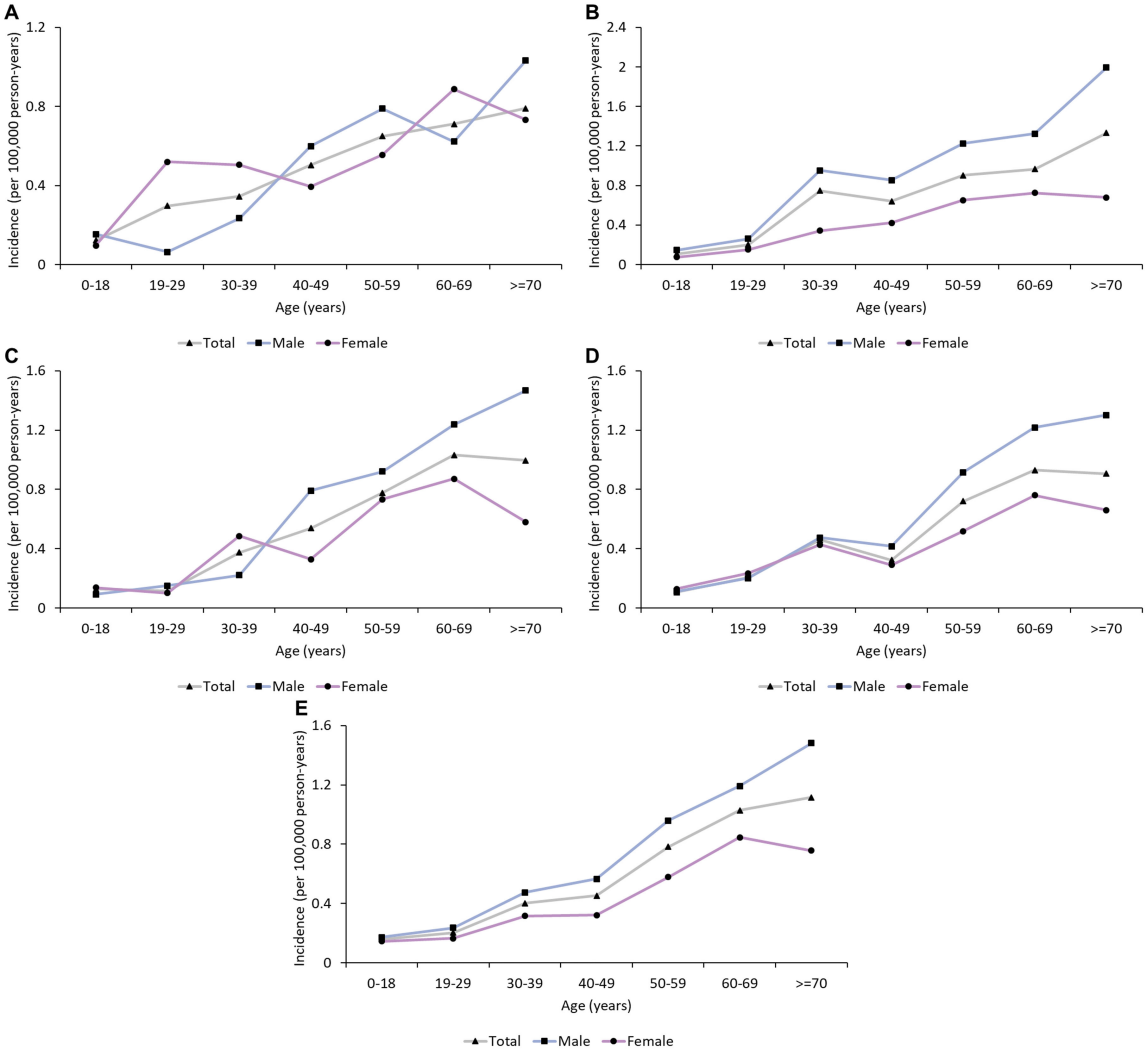
Age trend in the incidence of GBS in urban China (2013-2017). **(A)** 2013; **(B)** 2014; **(C)**: 2015; **(D)**: 2016; **(E)**: 2017. The incidence of GBS increased with age.

From 2013 to 2017, a total of 1.44 billion person-years was covered by UEBMI and URBMI, with 3,534 patients with GBS (2,137 males and 1,397 females) finally identified (**Table 1**). The annual incidences of GBS in 2013, 2014, 2015, 2016 and 2017 were 0.44 (95% CI: 0.25 to 0.67), 0.58 (95% CI: 0.38 to 0.82), 0.42 (95% CI: 0.28 to 0.58), 0.41 (95% CI: 0.27 to 0.58) and 0.51 (95% CI: 0.35 to 0.69) per 100,000 person-years, respectively. The results of subgroup analyses (sex, area, and age group) in different years during the study period were similar. The male incidence was higher than the female incidence (0.49 versus 0.35, rate ratio: 1.4, exact Poisson test: *P*<0.001) in 2017 (**Table 2**). In 2017, the incidence in Northwest China was the highest (0.87, 95% CI: 0.13 to 2.30, exact Poisson test: *P*<0.001), and the incidence in Northeast China was the lowest (0.26, 95% CI: 0.0002 to 1.00, exact Poisson test: *P*<0.001). The incidence increased with age (**Figure 1**). Stratified by season, the incidence was higher in summer (0.57, 95% CI: 0.38 to 0.79, exact Poisson test: *P*<0.001). The age-adjusted incidences of the entire population, male population and female population in 2017 were 0.45 (95% CI: 0.42 to 0.46), 0.55 (95% CI: 0.53 to 0.57), and 0.35 (95% CI: 0.33 to 0.36), respectively. The results of the sensitivity analysis can be seen in **Supplementary Data Sheet 6**, which was similar to the main results.

**Table 1:** Characteristics of patients with GBS in 23 provinces in urban China during 2013–2017.

Characteristics	Male (n=2,137)	Female (n=1,397)	Total (n=3,534)
**Mean age (years, SD)**	53.89 (17.86)	52.62 (19.02)	53.39 (18.34)
Age group, n (%)			
0 to 18	94 (4.40)	81 (5.80)	175 (4.95)
19 to 29	132 (6.18)	107 (7.66)	239 (6.76)
30 to 39	208 (9.73)	137 (9.81)	345 (9.76)
40 to 49	318(14.88)	203 (14.53)	521 (14.74)
50 to 59	477 (22.32)	290 (20.76)	767 (21.70)
60 to 69	466 (21.81)	307 (21.98)	773 (21.87)
≥ 70	442 (20.68)	272 (19.47)	714 (20.20)

GBS, Guillain-Barré syndrome; SD, standard deviation.

**Table 2:** Incidence of GBS in China in 2017 by sex, area, age group, and season.

Subgroup	Incidence (per 100,000 person-years, 95% CI)
Sex	
Male	0.49 (0.32 to 0.69)
Female	0.35 (0.23 to 0.50)
Area	
East	0.58 (0.40 to 0.79)
North	0.37 (0.29 to 0.47)
Northeast	0.26 (0.0002 to 1.00)
Northwest	0.87 (0.13 to 2.30)
Southcentral	0.52 (0.14 to 1.12)
Southwest	0.40 (0.02 to 1.26)
Age group	
0 to 18	0.16 (0.08 to 0.26)
19 to 29	0.20 (0.12 to 0.30)
30 to 39	0.40 (0.25 to 0.59)
40 to 49	0.45 (0.29 to 0.66)
50 to 59	0.78 (0.51 to 1.12)
60 to 69	1.03 (0.71 to 1.41)
≥ 70	1.12 (0.76 to 1.54)
Season	
Spring	0.46 (0.28 to 0.67)
Summer	0.57 (0.38 to 0.79)
Autumn	0.49 (0.33 to 0.69)
Winter	0.46 (0.32 to 0.63)

GBS, Guillain-Barré syndrome.

### 3.2 Systematic review and meta-analysis

**Figure 2:**
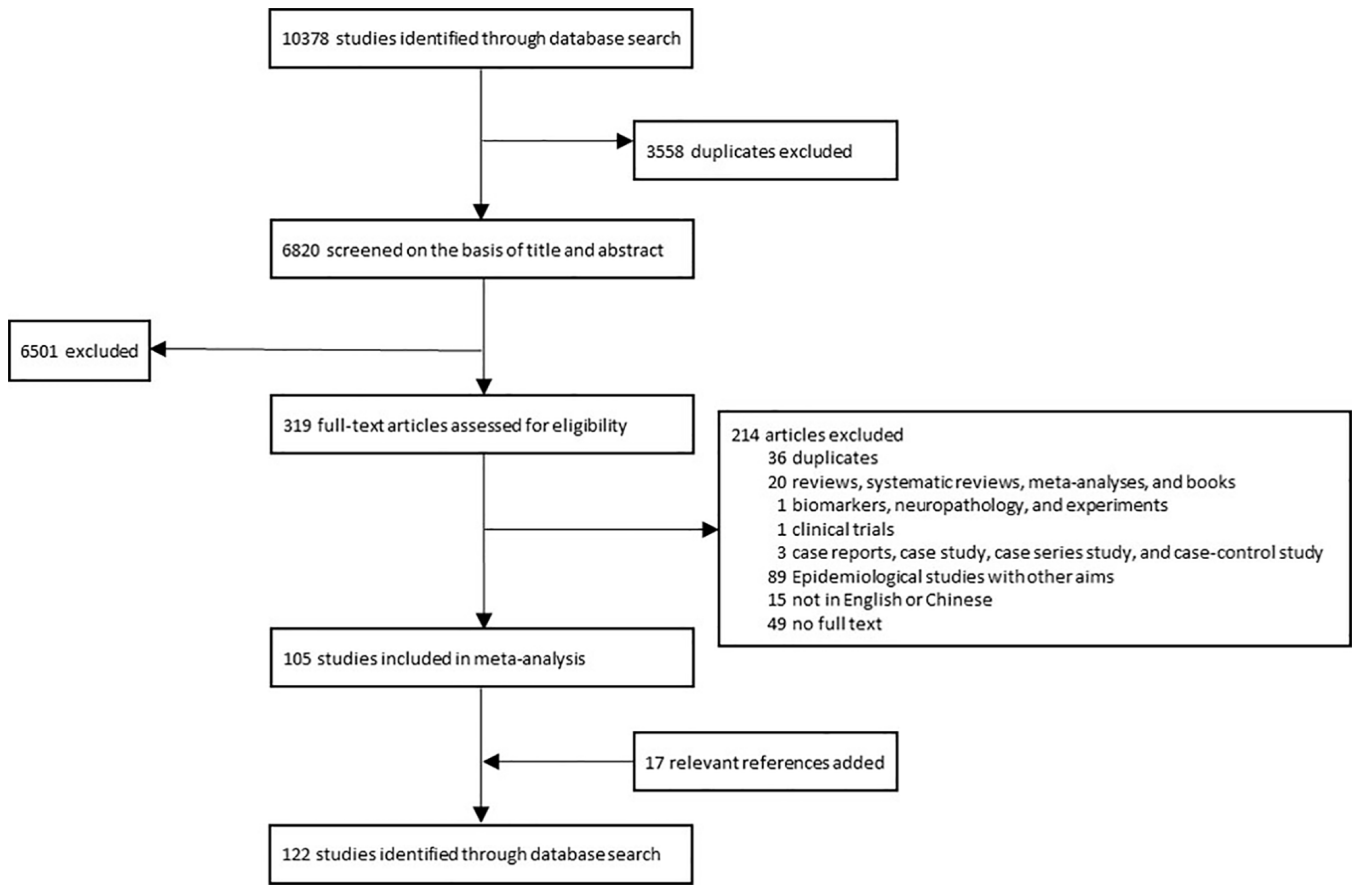
The process of selecting literatures for inclusion in systematic review and meta-analysis. A total of 122 studies were finally included in the systematic review and meta-analysis.

**Figure 3:**
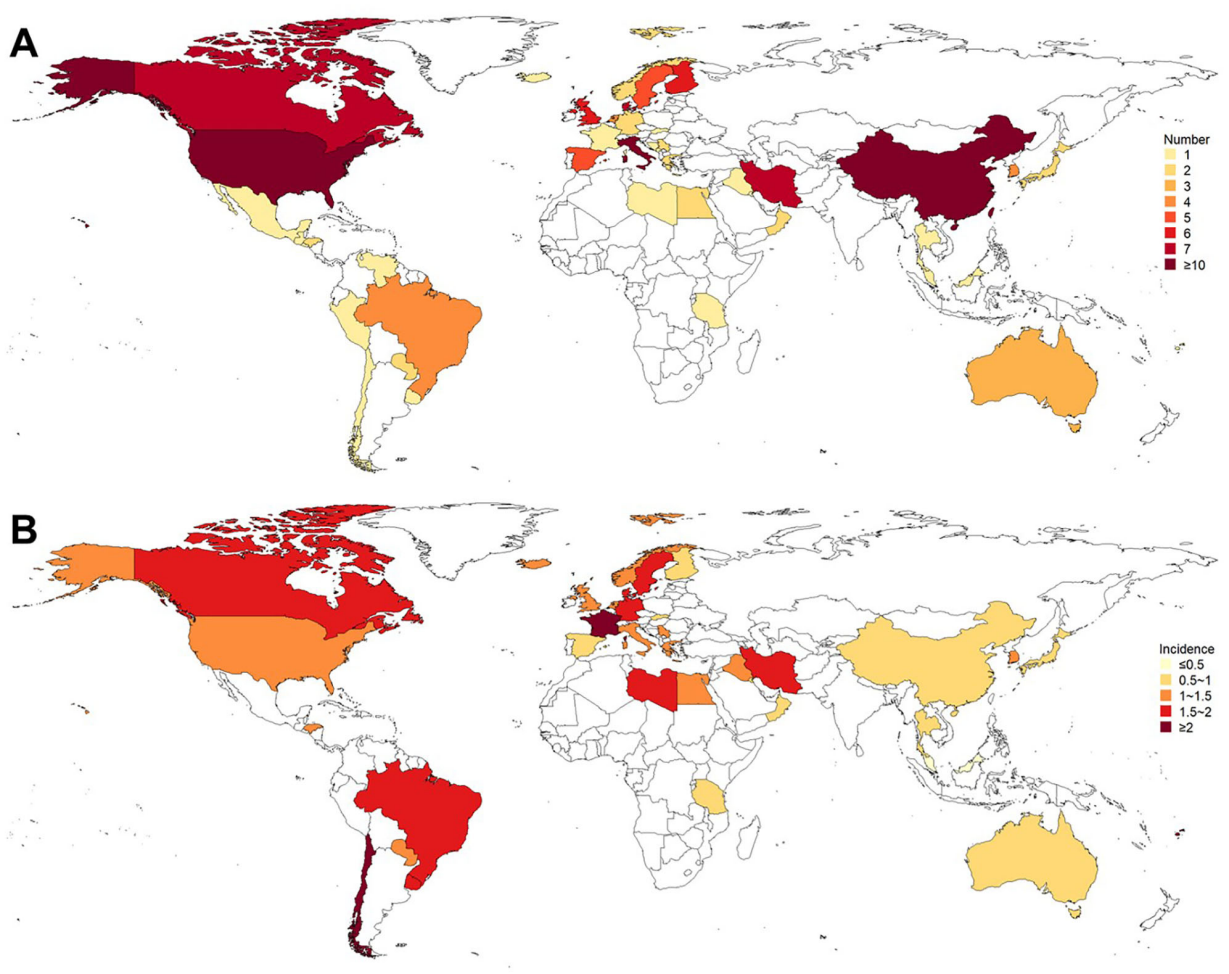
Existing population-based studies on the incidence of GBS. **(A)** Number of studies; **(B)** Incidence (per 100,000 person-years). Most of these studies were conducted in North Europe, South Europe, North America, and East Asia. The incidences in West Europe, South Asia, and North Europe were higher, while the incidences in Australia and New Zealand, Southeast Asia, and North Africa were lower.

A total of 122 articles were finally included through a literature search (**Figure 2**, **Supplementary Data Sheet 7**). The publication year spanned from 1976 to 2022. Most of these studies were conducted in North Europe (27 studies), South Europe (22 studies), North America (21 studies), and East Asia (16 studies) (**Figure 3**). The quality assessment of these articles showed that only 43.3% of studies had a quality score ≥ 0.75 (**Supplementary Data Sheet 8**).

**Figure 4:**
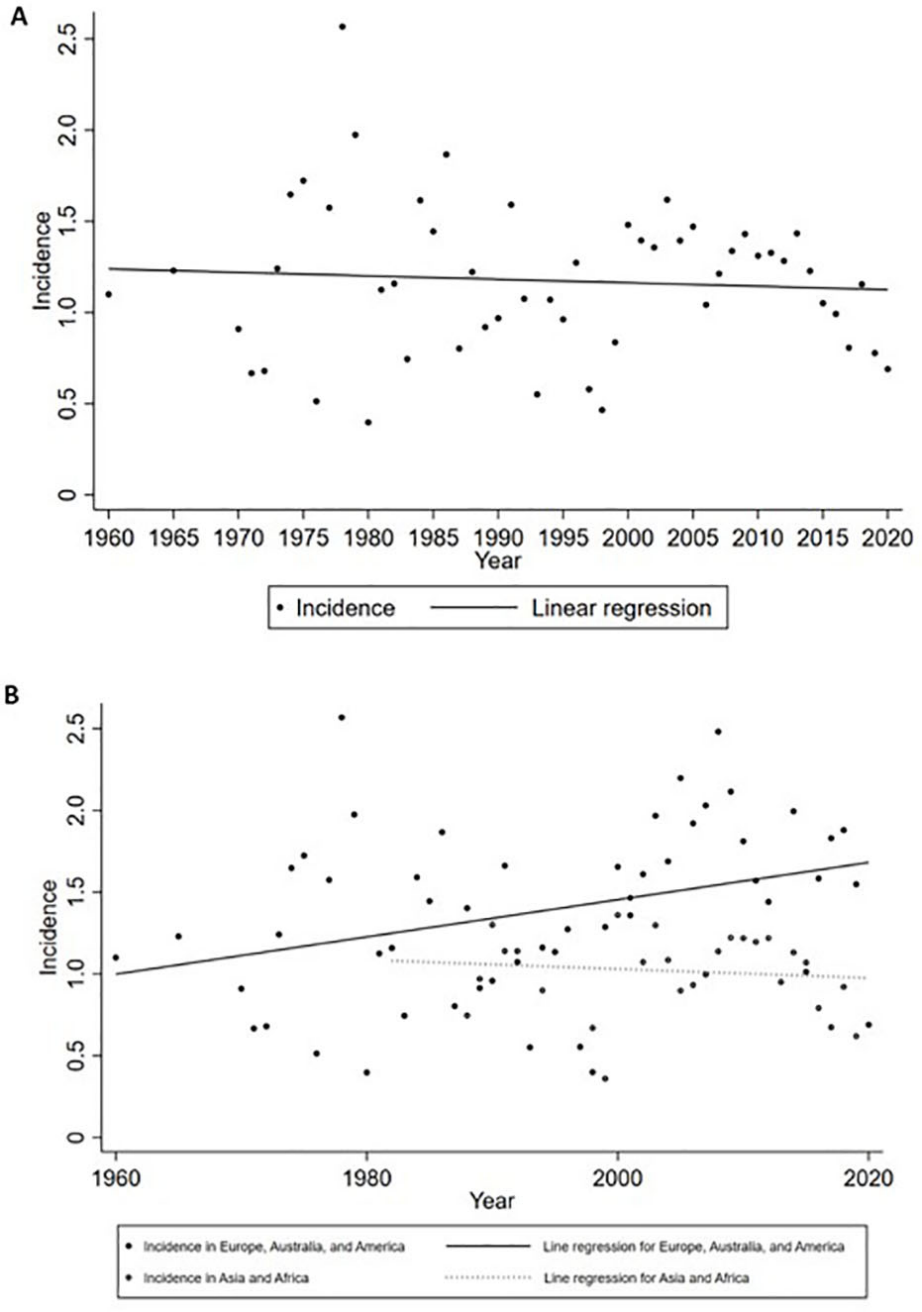
Incidence of GBS by year. **(A)** Global incidence; **(B)** Incidence grouped by subcontinents. No significant temporal trend was observed from 1960 to 2020 worldwide. When grouped by subcontinents, the incidence in Europe, Australia, and America rose significantly.

The global incidence of GBS was 1.12 per 100,000 person-years (95% CI: 0.98 to 1.27), with that of males and females was 1.38 per 100,000 person-years (95% CI: 1.14 to 1.64) and 0.99 per 100,000 person-years (95% CI: 0.81 to 1.18), respectively (**Table 3**). *I^2^* for combining all studies was 23.2%, indicating mild heterogeneity. Univariate meta-regression analysis identified heterogeneity in the subcontinent, study period, and study quality (**Supplementary Data Sheet 9**). The funnel plot and Egger’s test indicated publication bias (**Supplementary Data Sheet 10**). However, it may be worth noting that existing methods for detecting publication bias were developed for comparative data. They assume studies with positive results are published more often than those with negative results. This assumption is not necessarily true for a proportional meta-analysis, since there is no clear definition of the positive result (26). Therefore, the interpretation of the corresponding results needs to be cautious. The pooled incidence increased with age, from 0.48 (95% CI: 0.38 to 0.58) in those aged 0 to 18 years old to 1.59 (95% CI: 1.25 to 1.97) in those aged ≥ 70 years. The incidences in West Europe (1.92, 95% CI: 1.33 to 2.62), South Asia (1.61, 95% CI: 0.87 to 2.58), and North Europe (1.39, 95% CI: 1.04 to 1.80) were higher, while the incidences in Australia and New Zealand (0.54, 95% CI: 0.14 to 1.19), Southeast Asia (0.63, 95% CI 0.25 to 1.18), and North Africa (0.69, 95% CI: 0.01 to 1.81) were lower (**Table 3**, **Figure 3**). Countries with different economic levels showed no obvious difference in incidence. The incidences in spring (1.54, 95% CI: 0.55 to 3.03) and winter (1.72, 95% CI: 1.00 to 2.65) were higher. SDI, physician density, HDI, the proportion of the elderly population, and the incidence of respiratory infections were not observed to be associated with national GBS incidence (**Supplementary Data Sheet 11**), but the incidence of enteric infections was positively associated with GBS incidence (coefficient = 0.0000185, *P* = 0.007). No significant temporal trend was observed in GBS incidence from 1960 to 2020 (coefficient = -0.0018966, t = -0.52, *P* = 0.607, **Figure 4**) worldwide. However, when grouped by subcontinents, the incidence in Europe, Australia, and America rose significantly (coefficient = 0.01, t = 2.52, *P* = 0.015).

**Table 3:** Pooled incidence of GBS by sex, age, subcontinent, human development index, and season.

Subgroup	Incidence (per 100,000 person-years, 95% CI)
Sex	
Male	1.38 (1.14 to 1.64)
Female	0.99 (0.81 to 1.18)
Age	
0 to 18	0.48 (0.38 to 0.58)
19 to 29	0.41 (0.26 to 0.60)
30 to 39	0.54 (0.38 to 0.73)
40 to 49	0.68 (0.49 to 0.89)
50 to 59	1.08 (0.81 to 1.38)
60 to 69	1.40 (1.06 to 1.79)
≥ 70	1.59 (1.25 to 1.97)
Subcontinent	
West Europe	1.92 (1.33 to 2.62)
South Asia	1.61 (0.87 to 2.58)
North Europe	1.39 (1.04 to 1.80)
North America	1.38 (0.95 to 1.89)
South America	1.30 (0.52 to 2.42)
South Europe	0.97 (0.68 to 1.30)
East Asia	0.93 (0.71 to 1.18)
East Europe	0.89 (0.003 to 3.79)
West Asia	0.85 (0.18 to 2.02)
North Africa	0.69 (0.10 to 1.81)
Southeast Asia	0.63 (0.25 to 1.18)
Australia and New Zealand	0.54 (0.14 to 1.19)
Economic level	
Low-income economies	0.82 (0.30 to 1.59)
Lower-middle-income economies	1.24 (0.86 to 1.69)
Upper-middle-income economies	0.87 (0.55 to 1.25)
High-income economies	1.19 (1.02 to 1.37)
Season	
Spring	1.54 (0.55 to 3.03)
Summer	1.29 (0.81 to 1.90)
Autumn	1.18 (0.38 to 2.42)
Winter	1.72 (1.00 to 2.65)

GBS, Guillain-Barré syndrome.

## 4 Discussion

This study calculated the national incidence of GBS in urban China based on the population data from 2013 to 2017, and the annual incidence was between 0.41 and 0.58 per 100,000 person-years. In order to compare our national result with the global GBS burden, a meta-analysis was next conducted and elucidated a global incidence of 1.12 per 100,000 person-years. The GBS incidence increased with age and the male incidence was higher than that of females in urban China. These age and gender patterns were consistent with the results of our global meta-analysis and previous publications (1, 4, 12, 13, 27–29). Further investigation of the global meta-analysis revealed three important findings. First, the GBS incidence had clear regional differences. Second, the incidence of enteric infections was positively associated with national GBS incidences. Third, over the past decades, the incidence increased in Europe, Australia, and America, while it remained stable in other regions.

The annual GBS incidence in urban China was within the same range of a previous national study and a few provincial studies (12, 30, 31). In addition, our results showed that there was a clear regional difference in urban China, with the highest incidence in the Northwest and the lowest incidence in the Northeast. Zheng et al. also found that the northwest areas and southeast coastal areas had a higher incidence than other areas in China based on hospitalization case reports (12). From a global perspective, our meta-analysis showed clear regional differences at inter- and intra-continental levels. Specifically, West Europe, South Asia, and North Europe had higher incidences, whereas Australia and New Zealand, Southeast Asia, and North Africa had lower incidences. We further investigated the reasons behind regional variations by taking several factors into consideration. It has been proposed that healthcare infrastructure may potentially affect GBS incidences (32), but our analysis did not find an association of physician density with national GBS incidences. Further investigation of other factors of the healthcare system is required in the future. The proportion of the elderly population, HDI or SDI was not associated with national GBS incidence either. Although the GBD 2019 reported a positive association between GBS prevalence and SDI, a composite indicator of the development level, the association was not strong (4). These results suggest that other factors may primarily affect the regional variation of GBS incidences. Several antecedent infections have been associated with GBS, such as upper respiratory tract infection and gastroenteritis related to *Campylobacter jejuni* infection (1). We analyzed the contribution of these infections and found that the incidence of enteric infections was positively associated with national GBS incidence. Indeed, Wachira et al. conducted a systematic review and found that *Campylobacter jejuni* is the major trigger of GBS compared to other infectious and non-infectious etiological agents (33). This seems to be inconsistent with findings of the International Guillain-Barré Syndrome Outcome Study (IGOS), currently the largest global prospective observational cohort study (34). Recent results of IGOS showed that the preceding infections and their associated GBS subtypes varied among different regions (29), but the distribution of infections was similar across geographical regions (35). It is worth noting, however, that in IGOS the number of inclusions varied among regions and the majority of the patients were from temperate regions (35, 36). Al-Hakem et al. compared their national cohort with the Danish patients enrolled in IGOS (IGOS-DK) during the same period and found that the national cohort had a larger proportion of milder GBS cases (37). Therefore, patients enrolled in the IGOS cohort may not be the best representation of local or global populations (36, 37). Overall, our result suggests that variation in enteric infection underlies the regional difference in GBS incidences. This implies the importance of controlling these infections to reduce the GBS burden.

Seasonal fluctuation in incidence is another feature of GBS epidemiology, and our meta-analysis showed a trend of higher incidence in spring and winter. Due to the limited number of studies reporting GBS incidence in four seasons, our meta-analysis only included population-based studies which were primarily from Europe and North America. Our finding is consistent with a previous meta-analysis of studies reporting the seasonal distribution of GBS, which showed that the occurrences of GBS increased in winter in Western countries (38). It is worth noting that Webb et al. calculated the incidence rate ratios (IRRs) for each season versus the average of the other seasons (38), whereas our study directly calculated the incidence of each season. Hence, the papers recruited in these two meta-analyses were different. This may explain the lack of a significant peak in spring and winter in our study. Webb et al. also found a reverse trend in the Indian subcontinent and Latin America with increased GBS occurrences in summer (38). Differences in the seasonality of prodromal illnesses may account for this seasonal variation. The upper respiratory tract infection caused by *Mycoplasma pneumoniae* or *Haemophilus influenzae* underlies the higher winter incidence in Western countries (39–41), whereas preceding diarrhea caused by *C. jejuni* infection is associated with the summer peak in the Indian subcontinent and Latin America (42–45). Unfortunately, few papers from these tropical regions fulfill our requirement for calculating the incidence of each season. Therefore, more studies are required to clarify the seasonality feature of GBS incidence at the global level.

A wide range of papers enrolled in the meta-analysis allowed us to analyze the temporal trend of global GBS incidence. We found the incidence of GBS increased in Europe, Australia, and America, while remaining stable in other regions from 1960 to 2020. Previous papers analyzing the temporal trend of GBS incidence at national levels have reported an increased change in the United States (46) and South Korea (7, 27) or no change in Denmark (47), Netherlands (48), and the United States (49). However, these national studies cannot reflect the change at continental levels and their data hardly exceeded ten years. The increasing temporal trend in Europe, Australia, and America found in this study may be accounted by the rapid aging since the GBS incidence is higher in the elderly (4). Better recognition of the disease due to standard diagnosis protocols and guidelines may be another explanation for this temporal increase in Western countries compared to Asia and Africa (32, 50, 51). Moreover, diagnosis of GBS in Asia and Africa may be hampered by limited healthcare resources and well-trained personnel (32). It is worth noting that the number of publications is much lower in these regions compared to that in Europe, Australia, and America. The lack of well-designed epidemiological studies in these regions renders the true incidence of GBS largely unknown. Therefore, more studies are required to clearly elucidate the temporal trend of GBS incidence worldwide.

Our study has several limitations. First, only the medical terms and ICD-10 codes were used to identify GBS patients from the Chinese insurance database. However, the same approach has been widely used in previous high-quality epidemiological studies (4, 12, 52), and the GBS incidence calculated by our study is consistent with other national and regional studies in China (12, 30, 31). This supports the reliability of our methodology. Second, patients diagnosed in rural areas were not covered by the UEBMI and URBMI databases; therefore, the results in this study may only be generalizable to the urban population in China. It is worth noting, however, that the urban population accounts for over 60% of the whole Chinese population. Third, the time span of the national study was only 5 years, which may affect the discovery of the actual temporal trend of the GBS incidence in urban China. Further investigations are required to monitor the long-term temporal trend. Nevertheless, this study provided a nationwide analysis of the GBS incidence and its subgroup patterns in urban China. In addition, our meta-analysis updated and described the characteristics of global GBS incidence over the past 60 years. Although Wachira et al. conducted a systematic review of global GBS incidence recently, they did not perform a meta-analysis and the time span of their review was merely between 1985 and 2020 (53).

In light of the increasing incidence in Europe, Australia, and America found by our study, careful control of potential risk factors, particularly the preceding enteric infections, should be conducted to reduce the disease burden. Given the growing aging population in Asia and Africa, an increasing incidence of GBS may occur in the near future. Hence, rigorous monitoring and prevention of the disease should be carried out. Recognition of GBS should also be boosted in Asia and Africa, and more epidemiological studies should be conducted in these regions to accurately determine the background GBS incidence.

## Data Availability

The raw data supporting the conclusions of this article will be made available by the authors, without undue reservation.
