# Supplementary material for: Variation in worldwide incidence of Guillain-Barré syndrome: a population-based study in urban China and existing global evidence
Source: Front Immunol. 2024 Sep 10;15:1415986. doi: 10.3389/fimmu.2024.1415986 (PMC11420027; doi:10.3389/fimmu.2024.1415986)
Supplement: Supplementary file 1 [file DataSheet1.docx]

Supplementary Material

# Supplementary file 1. Comparison of the 8 excluded provinces and the 23 included provinces

| **Aspects** | **Factors** | ***P*** |
| --- | --- | --- |
| Education | Proportion of middle school and above in those aged ≥ 6 years old | 0.9857 |
| Urbanization | Proportion of urban population | 0.1272 |
|  | Urban area (km^2^) | 0.7542 |
|  | Urban population density (person/km^2^) | 0.5560 |
| Economic level | Regional GDP (100 million US dollars) | 0.5301 |
|  | Consumption level of urban residents (US dollars) | 0.0719 |
| Climate | Annual average temperature (℃) | 0.9367 |
|  | Annual average relative humidity （%） | 0.1090 |
|  | Annual precipitation in the provincial capital (mm) | 0.4018 |
|  | Annual sunlight hours in the provincial capital | 0.8547 |
| Environment | Total sewage discharge (10,000 tons) | 0.3638 |
|  | Sulfur dioxide (10,000 tons) | 0.1183 |
|  | Nitrogen oxide (10,000 tons) | 0.1362 |
|  | Smoke and dust (10,000 tons) | 0.5161 |
|  | Output of general industrial solid waste (10,000 tons) | 0.2898 |
|  | Output of hazardous waste (10,000 tons) | 0.1979 |
| Healthcare-related factors | Number of health institutions | 0.5104 |
|  | Number of hospitals | 0.2510 |
|  | Number of primary health institutions | 0.5535 |
|  | Number of health technicians | 0.2332 |
|  | Number of hospital beds in urban health institutions | 0.3845 |


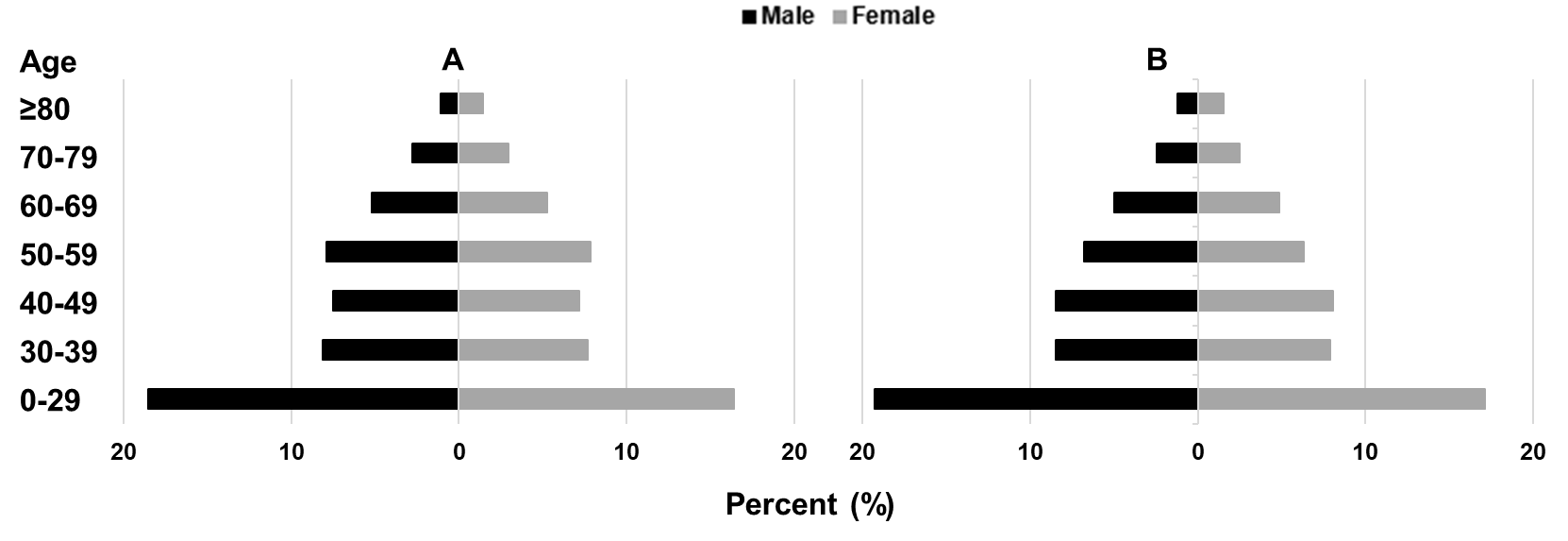


**Population Pyramid**

A: 2010 Chinese national census data; B: our study population

# Supplementary file 2. Keywords used to search for Guillain-Barré syndrome

| **Diagnostic name** | **Chinese characters** | **ICD-10** |
| --- | --- | --- |
| Guillain-Barré syndrome | 吉兰-巴雷综合征（症） | G61.002 |
| Guillain-Barré syndrome | 格林-巴利综合征（症） | G61.002 |
| acute inflammatory demyelinating polyradiculoneuropathy | 急性炎（症）性脱髓鞘性多发神经根神经病 | G61.004 |
| acute inflammatory demyelinating polyradiculoneuropathy | 急性炎症性脱髓鞘性多发神经根病 | G61.004 |
| acute inflammatory demyelinating polyradiculoneuropathy | 急性炎症性脱髓鞘性多发神经病 | G61.004 |
| acute motor axonal neuropathy | 急性运动轴索性神经病 | G61.003 |
| acute motor axonal neuropathy | 急性运动性（型）轴索性（型）神经病 | G61.003 |
| acute motor sensory axonal neuropathy | 急性运动感觉轴索性神经病 | G61.005 |
| acute motor sensory axonal neuropathy | 急性运动感觉性（型）轴索性（型）神经病 | G61.005 |
| Miller-Fisher syndrome | 费舍综合征（症） | G61.001 |
| Miller-Fisher syndrome | Miller-Fisher综合征（症） | G61.001 |
| Miller-Fisher syndrome | 米勒费雪综合征（症） | G61.001 |
| Miller-Fisher syndrome | 米勒费雪症候群 | G61.001 |
| Miller-Fisher syndrome | 米勒·费希尔综合征（症） | G61.001 |

# Supplementary file 3. Statistical method to estimate the incidence of Guillain-Barré syndrome

**Stage 1: Estimation of the incidence in each province**

The denominator used to calculate the rate of Guillain-Barré syndrome is defined as the total person-years in UEBMI and URBMI in each province during the corresponding period. In the pilot analysis for the national insurance database, we found that the diagnostic information may be missing, often due to administrative reasons. The total enrolled population in each subgroup (*N_i_*) can be classified into three categories: subjects with no records of any medical claim (*N_1i_*), subjects with complete information on diagnosis in claim records (*N_2i_*), and subjects with claim records but missing diagnostic information (*N_3i_*). Ignoring the GBS cases from *N_3i_* will lead to the underestimation of the incidence. Therefore, we design the following imputation algorithm to take account of the influence caused by missing information on the diagnosis.

The total population can be divided as shown in the following table.

|  | **Case** | **Non-case** | **Total** |
| --- | --- | --- | --- |
| Subjects with diagnostic information | *a_i_* | *b_i_* | *N_2i_* |
| Subjects without diagnostic information | *c_i_* | *d_i_* | *N_3i_* |
| Subjects without any claim | 0 | *e_i_* | *N_1i_* |
| **Total** | ***M_i_*** | ***N_i_ - M_i_*** | ***N_i_*** |

where *i* denoted the province. Patients with Guillain-Barré syndrome, noted as *M_i_*, are referred to the nominator to calculate the rate. Incidence can then be calculated as

$$\boldsymbol{I}_{\boldsymbol{i}}=\frac{\boldsymbol{a}_{\boldsymbol{i}}+\boldsymbol{c}_{\boldsymbol{i}}}{\boldsymbol{a}_{\boldsymbol{i}}+\boldsymbol{b}_{\boldsymbol{i}}+\boldsymbol{c}_{\boldsymbol{i}}+\boldsymbol{d}_{\boldsymbol{i}}+\boldsymbol{e}_{\boldsymbol{i}}} (A2)$$

Considering the fact that the missingness of diagnosis was often due to administrative reasons which should not be specific to Guillain-Barré syndrome, we assumed the proportion of Guillain-Barré syndrome cases in subjects with or without diagnostic information was equal, i.e.

$$\frac{\boldsymbol{a}_{\boldsymbol{i}}}{\boldsymbol{a}_{\boldsymbol{i}}+\boldsymbol{b}_{\boldsymbol{i}}}=\frac{\boldsymbol{c}_{\boldsymbol{i}}}{\boldsymbol{c}_{\boldsymbol{i}}+\boldsymbol{d}_{\boldsymbol{i}}} (A3)$$

The number of cases in subjects without diagnostic information (*c_ij_*) was then expressed as

$$\boldsymbol{c}_{\boldsymbol{i}}=\frac{\boldsymbol{a}}{N_{2i}}\times N_{3i}=r_{i}N_{3i} (A4)$$

**Stage 2: Pooling at the** **regional or national level**

The province-specific incidences were pooled using a random-effects meta-analysis to obtain the regional or national incidence. The Freeman-Tukey double arcsine transformation was used for stabilizing the variance of incidences at the provincial level.

# Supplementary file 4. Search strategy

(1) Medline

#1 "Guillain-Barré Syndrome"[Mesh] OR “Miller Fisher Syndrome” [Mesh]

#2 "Epidemiology"[Mesh] OR "Incidence"[Mesh] OR "epidemiology" [Subheading]

#3 “Guillain-Barré” OR “acute inflammatory demyelinating polyradiculoneuropathy” OR “acute motor axonal neuropathy” OR “acute motor sensory axonal neuropathy” OR “Miller Fisher”

#4 incidences OR incidence OR epidemiology OR epidemiologic*

#5 (#1 OR #3)

#6 (#2 OR #4)

#7 #5 AND #6

(2) Embase

#1 'Guillain-Barré' OR 'Guillain-Barré Syndrome'/exp OR 'Miller Fisher' OR 'Miller Fisher Syndrome'/exp OR 'acute inflammatory demyelinating polyradiculoneuropathy' OR 'acute motor axonal neuropathy' OR 'acute motor sensory axonal neuropathy'

#2 incidences OR 'incidence'/exp OR incidence OR 'epidemiology'/exp OR epidemiology OR epidemiologic*

#3 #1 AND #2

(3) Web of Science

#1 “Guillain-Barré” OR “Miller Fisher” OR “acute inflammatory demyelinating polyradiculoneuropathy” OR “acute motor axonal neuropathy” OR “acute motor sensory axonal neuropathy”

#2 incidences OR incidence OR epidemiology OR epidemiologic*

#3 #1 AND #2

# Supplementary file 5: Quality assessment tool

Articles are assigned 1 point for each “yes” received on the following scale for a total score out of 8. If a question is not applicable to the study under review, select “not applicable” so that the final quality denominator is lower (e.g., out of 7) for the given study.

**Representativeness of Sample**

1. Is the target population clearly defined?

□ Yes □ No □ Unclear □ Not reported

e.g. The target population must be defined by shared characteristics assessed and measured accurately. Some of these characteristics include age, sex, ethnicity, income, etc. Clear inclusion and exclusion criteria.

2. Was either of the following ascertainment methods used (must be one or the other)

1) probability sampling OR

2) entire population surveyed

□ Yes □ No □ Unclear □ Not reported

e.g. Members of the target population were identified through a sampling frame or listing of potential respondents. This listing must provide access to all members of the defined target population except for exclusions acknowledged by the study authors.

3. Is the response rate ≥70%

□ Yes □ No □ Not reported □ Not Applicable

4. Are non-responders clearly described?

□ Yes □ No □ Not reported □ Not Applicable

5. Is the sample representative of the target population?

□ Yes □ No □ Unclear □ Not reported

e.g. need to ensure that non-responders have similar characteristics as responders (otherwise may have selection bias)

**Assessment of Neurological Condition**

6. Were data collection methods standardized?

□ Yes □ No □ Unclear □ Not reported

e.g. Identical methods of assessment and data collection were used with all respondents so that the information for analysis is completely comparable. Standardization of methods not only refers to eliciting information from respondents but also to interviewing training, supervision, enlistment of respondents, and processing of data.

7. Were validated criteria used to assess for the presence/absence of disease?

□ Yes □ No □ Unclear □ Not reported

e.g. or a validated scale, diagnostic tool, survey, etc.

**Statistical Analysis**

8. Are the estimates of prevalence and incidence given with confidence intervals and in detail by subgroup (if applicable)?

□ Yes □ No □ Unclear □ Not reported

Quality Score Numerator: ____

Quality Score Denominator: ____

# Supplementary file 6. Sensitivity analysis of the incidence of Guillain-Barré syndrome in China in 2017 (units: per 100,000 person-years)

| **Reference population** | **Male (95% CI)** | **Female (95% CI)** | **Total (95% CI)** |
| --- | --- | --- | --- |
| **Incidence** |  |  |  |
| not imputing the numerator population from those without diagnostic information | 0.35 (0.24 to 0.48) | 0.25 (0.15 to 0.36) | 0.30 (0.20 to 0.42) |
| excluding the top 10% of provinces with missing diagnostic information | 0.60 (0.39 to 0.86) | 0.42 (0.26 to 0.61) | 0.52 (0.33 to 0.74) |

# Supplementary file 7. List of included studies

| No. | year | author | nation | subcontinent | eco_level | HDI | Incidence (per 100,000 person-years) | Standard error of incidence |
| --- | --- | --- | --- | --- | --- | --- | --- | --- |
| 1 | 1976 | Andersen, O. | Sweden | North Europe | High-income economies | Very high human development | 0.666667 | 0.210818 |
| 2 | 1979 | Hogg, J. E. | US | North America | High-income economies | Very high human development | 1.21 | 0.637722 |
| 3 | 1982 | Haberman, S. | UK | North Europe | High-income economies | Very high human development | 1.1 | 0.178173 |
| 4 | 1982 | Kaplan, J. E. | US | North America | High-income economies | Very high human development | 0.321 | 0.014463 |
| 5 | 1985 | Bak, P. | Denmark | North Europe | High-income economies | Very high human development | 1.1 | 0.663321 |
| 6 | 1985 | Larsen, J. P. | Norway | North Europe | High-income economies | Very high human development | 1.14 | 0.579043 |
| 7 | 1987 | Hankey, G. J. | Australia | Australia and New Zealand | High-income economies | Very high human development | 1.35 | 0.316931 |
| 8 | 1987 | Radhakrishnan, K. | Libya | North Africa | Upper-middle-income economies | Very high human development | 1.74 | 0.579995 |
| 9 | 1988 | Hallas, J. | Denmark | North Europe | High-income economies | Very high human development | 13.6 | 2.332222 |
| 10 | 1989 | Storey, E. | UK | Australia and New Zealand | High-income economies | Very high human development | 0.9 | 0.19188 |
| 11 | 1990 | Winner, S. J. | UK | North Europe | High-income economies | Very high human development | 1.1 | 0.467408 |
| 12 | 1991 | Farkkila, M. | Finland | North Europe | High-income economies | Very high human development | 1.1 | 0.307939 |
| 13 | 1991 | Koobatian, T. J. | US | North America | High-income economies | Very high human development | 1.6 | 0.548791 |
| 14 | 1991 | Paolino, E. | Italy | South Europe | High-income economies | Very high human development | 1.26 | 0.83434 |
| 15 | 1991 | Rantala, H. | Finland | North Europe | High-income economies | Very high human development | 0.38 | 0.079082 |
| 16 | 1993 | Matias-Guiu, J. | Spain | South Europe | High-income economies | Very high human development | 0.93 | 0.833081 |
| 17 | 1993 | Sridharan, G. V. | UK | North Europe | High-income economies | Very high human development | 1.1 | 0.450084 |
| 18 | 1994 | Hung, K. L. | China | East Asia | Low-income economies | Very high human development | 0.66 | 0.110615 |
| 19 | 1994 | Sedano, M. J. | Spain | South Europe | High-income economies | Very high human development | 0.95 | 0.428248 |
| 20 | 1995 | Jiang, G. X. | Sweden | North Europe | High-income economies | Very high human development | 1.74 | 0.17583 |
| 21 | 1995 | Kusumi, M. | Japan | East Asia | High-income economies | Very high human development | 1.14 | 0.429493 |
| 22 | 1996 | Beghi, E. | Italy | South Europe | High-income economies | Very high human development | 0.92 | 0.101717 |
| 23 | 1996 | Govoni, V. | Italy | South Europe | High-income economies | Very high human development | 1.87 | 1.027851 |
| 24 | 1997 | Emilia-Romagna Study Group on Clinical and Epidemiological Problems in Neurology | Italy | South Europe | High-income economies | High human development | 1.2 | 0.175209 |
| 25 | 1997 | Jiang, G. X. | Sweden | North Europe | High-income economies | Very high human development | 1.89 | 0.362155 |
| 26 | 1997 | Olivé, J. M. | |  |  |  |  | 9.10E-01 |
| 27 | 1997 | Prevots, D. R. | US | North America | High-income economies | Very high human development | 3 | 0.09248 |
| 28 | 1998 | Casmiro, M. | Italy | South Europe | High-income economies | High human development | 0.14 | 0.059845 |
| 29 | 1998 | Rees, J. H. | UK | North Europe | High-income economies | Very high human development | 1.2 | 0.13366 |
| 30 | 1999 | Govoni, V. | Italy | South Europe | High-income economies | Very high human development | 1.87 | 0.284889 |
| 31 | 2000 | Cheng, Q. | Sweden | North Europe | High-income economies | Very high human development | 1.63 | 0.190744 |
| 32 | 2000 | Hong, X. | China | East Asia | Low-income economies | Low human development | 0.9 | 0.137504 |
| 33 | 2000 | Van Koningsveld, R. | Netherlands | West Europe | High-income economies | Very high human development | 1.18 | 0.167615 |
| 34 | 2001 | Cuadrado, J. I. | Spain | South Europe | High-income economies | High human development | 0.85 | 0.046373 |
| 35 | 2001 | van Koningsveld, R. | Netherlands | West Europe | High-income economies | Very high human development | 2.53 | 1.285906 |
| 36 | 2002 | Cheng, Q. | China | East Asia | Lower-middle-income economies | Medium human development | 0.67 | 0.111813 |
| 37 | 1998 | Kinnunen, E. | Finland | North Europe | High-income economies | Very high human development | 0.82 | 0.128062 |
| 38 | 2002 | Dias-Tosta, E. | Brazil | South America | Upper-middle-income economies | Medium human development | 0.46 | 0.02971 |
| 39 | 2003 | Govoni, V. | Italy | South Europe | High-income economies | Very high human development | 1.91 | 0.239796 |
| 40 | 2005 | Hui, A. C. | Hong Kong | East Asia | High-income economies | Very high human development | 0.44 | 0.24205 |
| 41 | 2004 | Rocha, M. S. | Brazil | South America | Upper-middle-income economies | Medium human development | 0.4 | 0.041039 |
| 42 | 2002 | Varsik, P. | Slovakia | East Europe | Upper-middle-income economies | High human development | 0.8923 | 0.261989 |
| 43 | 2006 | Arami, M. A. | Iran | South Asia | Lower-middle-income economies | High human development | 2.11 | 0.242499 |
| 44 | 1996 | Howlett, W. P. | Norway | Sub-Saharan Africa | High-income economies | Very high human development | 0.83 | 0.108056 |
| 45 | 2006 | Hughes, R. A. | UK | North America | High-income economies | Very high human development | 1.411066 | 0.093449 |
| 46 | 2003 | Chiò, A. | Italy | South Europe | High-income economies | Very high human development | 1.36 | 0.127551 |
| 47 | 2000 | MacDonald, B. K. | UK | North Europe | High-income economies | Very high human development | 3 | 1.27551 |
| 48 | 2003 | Morris, A. M. S. | Australia | Australia and New Zealand | High-income economies | Very high human development | 0.38 | 0.046466 |
| 49 | 1994 | McLean, M. | Canada | North America | High-income economies | Very high human development | 2.07 | 0.057367 |
| 50 | 2003 | Potter, R. C. | US | North America | High-income economies | Very high human development | 0.63 | 0.082106 |
| 51 | 2007 | Lehmann, H. C. | | West Europe |  |  |  | 1.75E+00 |
| 52 | 2004 | Bogliun, G. | Italy | South Europe | High-income economies | Very high human development | 1.55 | 0.132027 |
| 53 | 2004 | Chroni, E. | Greece | South Europe | Upper-middle-income economies | High human development | 0.99 | 0.096939 |
| 54 | 2003 | Molinero, M. R. | Honduras | Latin America and the Caribbean | Low-income economies | Low human development | 1.37 | 0.211177 |
| 55 | 2004 | Cuadrado, J. I. | Spain | South Europe | High-income economies | Very high human development | 1.25 | 0.12618 |
| 56 | 1994 | Hart, D. E. | Paraguay | Latin America and the Caribbean | Lower-middle-income economies | Medium human development | 1.1 | 0.180838 |
| 57 | 2004 | Hussain, I. H. M. I. | Malaysia | Southeast Asia | Upper-middle-income economies | High human development | 0.36 | 0.06445 |
| 58 | 1985 | Beghi, E. | US | North America | High-income economies | Very high human development | 1.68 | 0.242237 |
| 59 | 1998 | Ismail, E. A. | Kuwait | North Africa | High-income economies | High human development | 0.95 | 0.232792 |
| 60 | 2004 | Zhang, Y. | China | East Asia | Low-income economies | Medium human development | 0.67 | 0.111275 |
| 61 | 1994 | Rantala, H. | US | North America | High-income economies | Very high human development | 0.6 | 0.063776 |
| 62 | 1997 | Jiang, G. X. | Sweden | North Europe | High-income economies | Very high human development | 1.77 | 0.037257 |
| 63 | 1979 | Schonberger, L. B. | US | North America | High-income economies | Very high human development | 0.432 | 0.020568 |
| 64 | 2007 | Barzegar, M. | Iran | South Asia | Lower-middle-income economies | High human development | 2.27 | 0.178571 |
| 65 | 2007 | Eaton, W. W. | Denmark | North Europe | High-income economies | Very high human development | 2.3 | 0.205015 |
| 66 | 2007 | Howitz, M. F. | Denmark | North Europe | High-income economies | Very high human development | 2 | 0.192093 |
| 67 | 2007 | Markoula, S. | Greece | South Europe | High-income economies | Very high human development | 1.22 | 0.552265 |
| 68 | 2008 | Alshekhlee, A. | US | North America | High-income economies | Very high human development | 1.65 | 0.143568 |
| 69 | 2008 | Deceuninck, G. | Canada | North America | High-income economies | Very high human development | 0.8 | 0.147959 |
| 70 | 2008 | Hauck, L. J. | Canada | North America | High-income economies | Very high human development | 1.6 | 0.201006 |
| 71 | 2008 | Koul, R. | Oman | West Asia | Upper-middle-income economies | High human development | 0.45 | 0.057522 |
| 72 | 2010 | Kang, J. H. | China | East Asia | Lower-middle-income economies | Medium human development | 0.4928 | 0.228114 |
| 73 | 2011 | Ruzante, J. M. | Canada | North America | High-income economies | Very high human development | 2.6 | 0.103833 |
| 74 | 2011 | van der Maas, N. A. | Netherlands | West Europe | High-income economies | Very high human development | 1.14 | 0.422376 |
| 75 | 2012 | Kandil, M. R. | Egypt | North Africa | Lower-middle-income economies | Medium human development | 12 | 5.357143 |
| 76 | 2012 | Lee, C. D. | US | North America | High-income economies | Very high human development | 1.4 | 0.09949 |
| 77 | 2012 | Shui, I. M. | US | North America | High-income economies | Very high human development | 3.13 | 0.07889 |
| 78 | 2013 | Jasem, J. | Iraq | West Asia | Lower-middle-income economies | Medium human development | 1.33 | 0.181122 |
| 79 | 2014 | Chen, Y. | China | East Asia | Lower-middle-income economies | Medium human development | 0.59 | 0.048662 |
| 80 | 2014 | Hense, S. | Germany | West Europe | High-income economies | Very high human development | 2.6 | 0.139826 |
| 81 | 2014 | Peric, S. |  |  |  |  | 0.87 | 0.043367 |
| 82 | 2015 | Benedetti, M. D. | Italy | South Europe | High-income economies | Very high human development | 1.84 | 0.096287 |
| 83 | 2015 | Huang, W. C. | China | East Asia | Lower-middle-income economies | Medium human development | 1.65 | 0.082513 |
| 84 | 2015 | Sipila, J. O. T. | Finland | North Europe | High-income economies | Very high human development | 1.82 | 0.692054 |
| 85 | 2016 | Liou, L. S. | China | East Asia | Lower-middle-income economies | Medium human development | 1.71E+00 | 0.023104 |
| 86 | 2016 | Momen, A. A. | Iran | South Asia | Lower-middle-income economies | High human development | 1.95 | 0.403109 |
| 87 | 2016 | Rivera-Lillo, G. | Chile | South America | Upper-middle-income economies | High human development | 2.12 | 0.104229 |
| 88 | 2016 | Suryapranata, F. S. T. | Netherlands | West Europe | High-income economies | Very high human development | 3.93 | 1.925461 |
| 89 | 2017 | Delannoy, A. | France | West Europe | High-income economies | Very high human development | 2.42 | 0.061169 |
| 90 | 2017 | Momen, A. A. | Iran | South Asia | Upper-middle-income economies | High human development | 1.51 | 0.34756 |
| 91 | 2017 | Pastula, D. M. | Fiji | Melanesia | Lower-middle-income economies | High human development | 5 | 0.763362 |
| 92 | 2017 | Salinas, J. L. | Puerto Rico | North America | High-income economies | Very high human development | 3.8 | 0.651682 |
| 93 | 2017 | Sipila, J. O. T. | Finland | North Europe | High-income economies | Very high human development | 1.69 | 0.17823 |
| 94 | 2017 | Skufca, J. | Finland | North Europe | High-income economies | Very high human development | 2.59 | 0.389176 |
| 95 | 2018 | Auger, N. | Canada | North America | High-income economies | Very high human development | 1.42 | 0.093887 |
| 96 | 2018 | Hafsteinsdóttir, B. | Iceland | North Europe | High-income economies | Very high human development | 1.1 | 0.619776 |
| 97 | 2018 | Matsui, N. | Japan | East Asia | High-income economies | Very high human development | 0.42 | 0.167332 |
| 98 | 2019 | Abdel-Fattah, A. | Egypt | North Africa | Lower-middle-income economies | Medium human development | 0.3538 | 0.120764 |
| 99 | 2019 | Al-Hakem, H. | Denmark | North Europe | High-income economies | Very high human development | 1.59 | 0.183903 |
| 100 | 2019 | Benedetti, L. | Italy | South Europe | High-income economies | Very high human development | 3 | 1.166372 |
| 101 | 2019 | Granieri, E. | Italy | South Europe | High-income economies | Very high human development | 1.38 | 0.625244 |
| 102 | 2019 | Koul, R. | Oman | West Asia | High-income economies | High human development | 3.1 | 0.566188 |
| 103 | 2019 | Levison, L. S. | Denmark | North Europe | High-income economies | Very high human development | 1.77 | 0.176216 |
| 104 | 2019 | Momen, A. A. | Iran | South Asia | Upper-middle-income economies | High human development | 1.545 | 0.358868 |
| 105 | 2020 | Lee, H. | South Korea | East Asia | High-income economies | Very high human development | 4.15 | 0.311341 |
| 106 | 2020 | Leung, J. | US | North America | High-income economies | Very high human development | 1.25 | 0.056013 |
| 107 | 2020 | Levison, L. S. | Denmark | North Europe | High-income economies | Very high human development | 0.69 | 0.262678 |
| 108 | 2020 | Malta, J. M. A. S. | Brazil | South America | Upper-middle-income economies | High human development | 28.4 | 0.720979 |
| 109 | 2020 | Stojanov, A. | Serbia | South Europe | Upper-middle-income economies | High human development | 1.07 | 0.133749 |
| 110 | 2021 | Carey, I. M. | UK | North Europe | High-income economies | Very high human development | 1.71 | 0.053037 |
| 111 | 2021 | Chiesa, M. | Uruguay | South America | High-income economies | Very high human development | 1.7 | 0.346856 |
| 112 | 2021 | Filosto, M. | Italy | South Europe | High-income economies | Very high human development | 0.93 | 0.257934 |
| 113 | 2021 | Fragiel, M. | Spain | South Europe | High-income economies | Very high human development | 0.69 | 0.120113 |
| 114 | 2021 | Kasemsap, N. | Thailand | Southeast Asia | Upper-middle-income economies | High human development | 0.8 | 0.035049 |
| 115 | 2021 | Kim, A. Y. | South Korea | East Asia | High-income economies | Very high human development | 1.48 | 0.153823 |
| 116 | 2021 | Tonekaboni, S. H. | Iran | South Asia | Upper-middle-income economies | High human development | 1.72 | 0.098627 |
| 117 | 2021 | Wachira, V. K. | Brazil | South America | Upper-middle-income economies | High human development | 0.74 | 0.018694 |
| 118 | 2021 | Yoon, H. H. | South Korea | East Asia | High-income economies | Very high human development | 1.25 | 0.115872 |
| 119 | 2022 | Hosseininezhad, M. | Iran | South Asia | Upper-middle-income economies | High human development | 0.69 | 0.165414 |
| 120 | 2022 | Nasreen, S. | Canada | North America | High-income economies | Very high human development | 1.86 | 0.115263 |
| 121 | 2022 | Yi, S. W. | South Korea | East Asia | High-income economies | Very high human development | 1.29 | 0.050737 |
| 122 | 2022 | Zheng, P. | China | East Asia | Upper-middle-income economies | High human development | 0.7 | 0.007102 |

1. Andersen O, Eeg Olofsson O. A prospective study of parapareses in Western Sweden. Acta Neurologica Scandinavica 1976; 54(4): 312-20.

2. Hogg JE, Kobrin DE, Schoenberg BS. GUILLAIN-BARRE-SYNDROME EPIDEMIOLOGIC AND CLINICAL-FEATURES. Journal of Chronic Diseases 1979; 32(3): 227-31.

3. Haberman S, Benjamin B, Capildeo R, Rose FC. North West Thames registry of neurological disease. J R Soc Med 1982; 75(6): 443-9.

4. Kaplan JE, Katona P, Hurwitz ES, Schonberger LB. Guillain-Barre syndrome in the United States, 1979-1980 and 1980-1981. Journal of the American Medical Association 1982; 248(6): 698-700.

5. Bak P. Guillain-Barre syndrome in a Danish county. Neurology 1985; 35(2): 207-11.

6. Larsen JP, Kvale G, Nyland H. Epidemiology of the Guillain-Barre syndrome in the county of Hordaland, Western Norway. Acta Neurologica Scandinavica 1985; 71(1): 43-7.

7. Hankey GJ. Guillain-Barre syndrome in western Australia, 1980-1985. Medical Journal of Australia 1987; 146(3): 130-3.

8. Radhakrishnan K, El-Mangoush MA, Gerryo SE. Descriptive epidemiology of selected neuromuscular disorders in Benghazi, Libya. Acta Neurologica Scandinavica 1987; 75(2): 95-100.

9. Hallas J, Bredkjaer C, Friis ML. GUILLAIN-BARRE-SYNDROME - DIAGNOSTIC-CRITERIA, EPIDEMIOLOGY, CLINICAL COURSE AND PROGNOSIS. Acta Neurologica Scandinavica 1988; 78(2): 118-22.

10. Storey E, Cook M, Peppard R, Newton-John H, Byrne E. Guillain-Barré syndrome and related conditions in Victorian teaching hospitals 1980-84. Aust N Z J Med 1989; 19(6): 687-93.

11. Winner SJ, Evans JG. AGE-SPECIFIC INCIDENCE OF GUILLAIN-BARRE-SYNDROME IN OXFORDSHIRE. Quarterly Journal of Medicine 1990; 77(284): 1297-304.

12. Farkkila M, Kinnunen E, Weckstrom P. Survey of Guillain-Barre syndrome in southern Finland. Neuroepidemiology 1991; 10(5-6): 236-41.

13. Koobatian TJ, Birkhead GS, Schramm MM, Vogt RL. THE USE OF HOSPITAL DISCHARGE DATA FOR PUBLIC-HEALTH SURVEILLANCE OF GUILLAIN-BARRE-SYNDROME. Annals of Neurology 1991; 30(4): 618-21.

14. Paolino E, Govoni V, Tola MR, Casetta I, Granieri E. INCIDENCE OF THE GUILLAIN-BARRE-SYNDROME IN FERRARA, NORTHERN ITALY, 1981-1987. Neuroepidemiology 1991; 10(3): 105-11.

15. Rantala H, Uhari M, Niemela M. OCCURRENCE, CLINICAL MANIFESTATIONS, AND PROGNOSIS OF GUILLAIN-BARRE-SYNDROME. Archives of Disease in Childhood 1991; 66(6): 706-8.

16. Matias-Guiu J, Martin R, Blanquer J, et al. Incidence of Guillain-Barre syndrome and ganglioside intake in Alcoi, Spain. Neuroepidemiology 1993; 12(1): 58-60.

17. Sridharan GV, Tallis RC, Gautam PC. Guillain-Barré syndrome in the elderly. A retrospective comparative study. Gerontology 1993; 39(3): 170-5.

18. Hung KL, Wang HS, Liou WY, et al. Guillain-Barré syndrome in children: a cooperative study in Taiwan. Brain Dev 1994; 16(3): 204-8.

19. Sedano MJ, Calleja J, Canga E, Berciano J. Guillain-Barre syndrome in Cantabria, Spain. An epidemiological and clinical study. Acta Neurologica Scandinavica 1994; 89(4): 287-92.

20. Jiang GX, De Pedro-Cuesta J, Fredrikson S. Guillain-Barre syndrome in South-West Stockholm, 1973-1991, 1. Quality of registered hospital diagnoses and incidence. Acta Neurologica Scandinavica 1995; 91(2): 109-17.

21. Kusumi M, Nakashima K, Nakayama H, Takahashi K. Epidemiology of inflammatory neurological and inflammatory neuromuscular diseases in Tottori Prefecture, Japan. Psychiatry and Clinical Neurosciences 1995; 49(3): 169-74.

22. Beghi E, Bogliun G. The Guillain-Barrè syndrome (GBS). Implementation of a register of the disease on a nationwide basis. Italian GBS Study Group. Italian journal of neurological sciences 1996; 17(5): 355-61.

23. Govoni V, Granieri E, Casetta I, et al. The incidence of Guillain-Barre syndrome in Ferrara, Italy: Is the disease really increasing? Journal of the Neurological Sciences 1996; 137(1): 62-8.

24. Neurology E-RSGoCaEPi. A prospective study on the incidence and prognosis of Guillain-Barré syndrome in Emilia-Romagna region, Italy (1992-1993). Emilia-Romagna Study Group on Clinical and Epidemiological Problems in Neurology. Neurology 1997; 48(1): 214-21.

25. Jiang GX, Cheng Q, Ehrnst A, Link H, De Pedro-Cuesta J. Guillain-Barre syndrome in Stockholm County, 1973-1991. European Journal of Epidemiology 1997; 13(1): 25-32.

26. Olivé JM, Castillo C, Castro RG, De Quadros CA. Epidemiologic study of Guillain-Barre syndrome in children <15 years of age in Latin America. Journal of Infectious Diseases 1997; 175(2 SUPPL.): S160-S4.

27. Prevots DR, Sutter RW. Assessment of Guillain-Barre syndrome mortality and morbidity in the United States: Implications for acute flaccid paralysis surveillance. Journal of Infectious Diseases 1997; 175(2 SUPPL.): S151-S5.

28. Casmiro M, Guarino M, D'Alessandro R, et al. Guillain-Barre syndrome variants in Emilia-Romagna, Italy, 1992-3: Incidence, clinical features, and prognosis. Journal of Neurology Neurosurgery and Psychiatry 1998; 65(2): 218-24.

29. Rees JH, Thompson RD, Smeeton NC, Hughes RAC. Epidemiological study of Guillain-Barre syndrome in south east England. Journal of Neurology Neurosurgery and Psychiatry 1998; 64(1): 74-7.

30. Govoni V, Granieri E, Tola MR, Casetta I, Ruppi P, Vaghi L. The frequency of clinical variants of Guillain-Barre syndrome in Ferrara, Italy. Journal of Neurology 1999; 246(11): 1010-4.

31. Cheng Q, Jiang GX, Fredrikson S, et al. Incidence of Guillain-Barré syndrome in Sweden 1996. European Journal of Neurology 2000; 7(1): 11-6.

32. Hong X, Zhang Z, Wang J, et al. Epidemiological characteristics of Guillain-Barré syndrome in urban and rural areas in Beijing and Hebei, China. Zhongguo yi xue ke xue yuan xue bao Acta Academiae Medicinae Sinicae 2000; 22(2): 115-9.

33. Van Koningsveld R, Van Doorn PA, Schmitz PIM, Ang CW, Van Der Meché FGA. Mild forms of Guillain-Barre syndrome in an epidemiologic survey in the Netherlands. Neurology 2000; 54(3): 620-5.

34. Cuadrado JI, de Pedro-Cuesta J, Ara JR, et al. Guillain-Barre syndrome in Spain, 1985-1997: Epidemiological and public health views. European Neurology 2001; 46(2): 83-91.

35. van Koningsveld R, Rico R, Gerstenbluth I, et al. Gastroenteritis-associated Guillain-Barre syndrome on the Caribbean island Curacao. Neurology 2001; 56(11): 1467-72.

36. Cheng Q, Huang DS, Jiang GX, et al. Distinct pattern of age-specific incidence of Guillain-Barre syndrome in Harbin, China. Psychosomatic Medicine 2002; 249(1):25-32.

37. Dias-Tosta E, Kückelhaus CS. Guillain Barré syndrome in a population less than 15 years old in Brazil. Arquivos de neuro-psiquiatria 2002; 60(2 B): 367-73.

38. Varsik P, Traubner P, Cernacek J, Traubnerova R. Influence of geomedical factors on Guillain-Barré syndrome incidence in the region of western Slovakia. Bratislavské lekárske listy 2002; 103(1): 30-3.

39. Chiò A, Cocito D, Leone M, et al. Guillain-Barré syndrome: A prospective, population-based incidence and outcome survey. Neurology 2003; 60(7): 1146-50.

40. Morris AMS, Elliott EJ, D'Souza RM, Antony J, Kennett M, Longbottom H. Acute flaccid paralysis in Australian children. Journal of Paediatrics and Child Health 2003; 39(1): 22-6.

41. Potter RC, Kaneene JB. A descriptive study of Guillain-Barré syndrome in high and low Campylobacter jejuni incidence regions of Michigan: 1992-1999. Neuroepidemiology 2003; 22(4): 245-8.

42. Bogliun G, Beghi E, Italian GBSRSG. Incidence and clinical features of acute inflammatory polyradiculoneuropathy in Lombardy, Italy, 1996. Acta Neurologica Scandinavica 2004; 110(2): 100-6.

43. Chroni E, Papapetropoulos S, Gioldasis G, Ellul J, Diamadopoulos N, Papapetropoulos T. Guillain-Barré syndrome in Greece: seasonality and other clinico-epidemiological features. Eur J Neurol 2004; 11(6): 383-8.

44. Cuadrado JI, de Pedro-Cuesta J, Ara JR, et al. Public health surveillance and incidence of adulthood Guillain-Barré syndrome in Spain, 1998-1999: The view from a sentinel network of neurologists. Neurological Sciences 2004; 25(2): 57-65.

45. Hussain IHMI, Ali S, Sinniah M, et al. Five-year surveillance of acute flaccid paralysis in Malaysia. Journal of Paediatrics and Child Health 2004; 40(3): 127-30.

46. Zhang Y, Wang DS, Han H, Li F, Sheng L, Link H. Epidemiological survey of the incidence of Guillain-Barré syndrome in Harbin from 1997 to 1999. Chinese Journal of Clinical Rehabilitation 2004; 8(34): 7812-5.

47. Barzegar M, Dastgiri S, Karegarmaher MH, Varshochiani A. Epidemiology of childhood Guillan-Barre syndrome in the north west of Iran. BMC Neurology 2007; 7.

48. Eaton WW, Rose NR, Kalaydjian A, Pedersen MG, Mortensen PB. Epidemiology of autoimmune diseases in Denmark. Journal of Autoimmunity 2007; 29(1): 1-9.

49. Howitz MF, Mølbak K. Campylobacter, polyneuropathy, and Guillain-Barré syndrome in Denmark, 1994-2003. Scandinavian Journal of Infectious Diseases 2007; 39(2): 160-2.

50. Markoula S, Giannopoulos S, Sarmas I, Tzavidi S, Kyritsis AP, Lagos G. Guillain-Barré syndrome in northwest Greece. Acta Neurologica Scandinavica 2007; 115(3): 167-73.

51. Alshekhlee A, Hussain Z, Sultan B, Katirji B. Guillain-Barré syndrome: Incidence and mortality rates in US hospitals. Neurology 2008; 70(18): 1608-13.

52. Deceuninck G, Boucher RM, de Wals P, Ouakki M. Epidemiology of Guillain-Barré syndrome in the province of Quebec. Canadian Journal of Neurological Sciences 2008; 35(4): 472-5.

53. Hauck LJ, White C, Feasby TE, Zochodne DW, Svenson LW, Hill MD. Incidence of Guillain-Barré syndrome in Alberta, Canada: An administrative data study. Journal of Neurology, Neurosurgery and Psychiatry 2008; 79(3): 318-20.

54. Koul R, Al-Futaisi A, Chacko A, et al. Clinical characteristics of childhood guillain-barré syndrome. Oman Med J 2008; 23(3): 158-61.

55. Kang J-H, Sheu J-J, Lin H-C. Increased Risk of Guillain-Barre Syndrome following Recent Herpes Zoster: A Population-Based Study across Taiwan. Clinical Infectious Diseases 2010; 51(5): 525-30.

56. Ruzante JM, Majowicz SE, Fazil A, Davidson VJ. Hospitalization and deaths for select enteric illnesses and associated sequelae in Canada, 2001-2004. Epidemiology and Infection 2011; 139(6): 937-45.

57. van der Maas NA, Kramer MA, Jacobs BC, et al. Guillain-Barré syndrome: background incidence rates in The Netherlands. J Peripher Nerv Syst 2011; 16(3): 243-9.

58. Kandil MR, Darwish ES, Khedr EM, Sabry MM, Abdulah MA. A community-based epidemiological study of peripheral neuropathies in Assiut, Egypt. Neurological Research 2012; 34(10): 960-6.

59. Lee CD, Jones TF. Hospital discharge database optimization in guillain-barré syndrome surveillance. Muscle and Nerve 2012; 46(1): 60-2.

60. Shui IM, Rett MD, Weintraub E, et al. Guillain-Barré syndrome incidence in a large United States cohort (2000-2009). Neuroepidemiology 2012; 39(2): 109-15.

61. Jasem J, Marof K, Nawar A, et al. Guillain-Barré syndrome as a cause of acute flaccid paralysis in Iraqi children: A result of 15 years of nation-wide study. BMC Neurology 2013; 13.

62. Chen Y, Ma F, Zhang J, Chu X, Xu Y. Population incidence of Guillain-Barré syndrome in parts of China: Three large populations in Jiangsu province, 2008-2010. European Journal of Neurology 2014; 21(1): 124-9.

63. Hense S, Schink T, Kreisel SH, et al. Estimation of background incidence rates of guillain-barré syndrome in Germany-A retrospective cohort study with electronic healthcare data. Neuroepidemiology 2014; 43(3-4): 244-52.

64. Peric S, Milosevic V, Berisavac I, et al. Clinical and epidemiological features of Guillain-Barré syndrome in the Western Balkans. Journal of the Peripheral Nervous System 2014; 19(4): 317-21.

65. Benedetti MD, Pugliatti M, Dalessandro R, et al. A Multicentric Prospective Incidence Study of Guillain-Barre Syndrome in Italy. the ITANG Study. Neuroepidemiology 2015; 45(2): 90-9.

66. Huang WC, Lu CL, Chen SCC. A 15-year nationwide epidemiological analysis of Guillain-Barré syndrome in Taiwan. Neuroepidemiology 2015; 44(4): 249-54.

67. Sipilä JOT, Soilu-Hänninen M. The incidence and triggers of adult-onset Guillain-Barré syndrome in southwestern Finland 2004-2013. European Journal of Neurology 2015; 22(2): 292-8.

68. Liou LS, Chung CH, Wu YT, et al. Epidemiology and prognostic factors of inpatient mortality of Guillain-Barré syndrome: A nationwide population study over 14 years in Asian country. Journal of the Neurological Sciences 2016; 369: 159-64.

69. Momen AA, Shakurnia A. An epidemiological analysis of acute flaccid paralysis in Khuzestan Province, southwest Iran, from 2006 to 2010. Epidemiology and health 2016; 38: e2016030.

70. Rivera-Lillo G, Torres-Castro R, Burgos PI, et al. Incidence of Guillain-Barré syndrome in Chile: a population-based study. Journal of the Peripheral Nervous System 2016; 21(4): 339-44.

71. Suryapranata FST, Ang CW, Chong LL, Murk JL, Falconi J, Huits RMHG. Epidemiology of Guillain-Barré syndrome in Aruba. American Journal of Tropical Medicine and Hygiene 2016; 94(6): 1380-4.

72. Delannoy A, Rudant J, Chaignot C, Bolgert F, Mikaeloff Y, Weill A. Guillain-Barré syndrome in France: a nationwide epidemiological analysis based on hospital discharge data (2008-2013). J Peripher Nerv Syst 2017; 22(1): 51-8.

73. Momen AA, Shakurnia A. The epidemiology of guillain-Barré Syndrome in children under 15 years old in Southwest Iran. Biomedicine Hub 2017; 2(3).

74. Pastula DM, Khan AS, Sharp TM, et al. Investigation of a Guillain-Barré syndrome cluster in the Republic of Fiji. Journal of the Neurological Sciences 2017; 372: 350-5.

75. Salinas JL, Major CG, Pastula DM, et al. Incidence and clinical characteristics of Guillain-Barré syndrome before the introduction of Zika virus in Puerto Rico. Journal of the Neurological Sciences 2017; 377: 102-6.

76. Sipilä JOT, Soilu-Hänninen M, Ruuskanen JO, Rautava P, Kytö V. Epidemiology of Guillain-Barré syndrome in Finland 2004–2014. Journal of the Peripheral Nervous System 2017; 22(4): 440-5.

77. Skufca J, Ollgren J, Ruokokoski E, Lyytikäinen O, Nohynek H. Incidence rates of Guillain Barré (GBS), chronic fatigue/systemic exertion intolerance disease (CFS/SEID) and postural orthostatic tachycardia syndrome (POTS) prior to introduction of human papilloma virus (HPV) vaccination among adolescent girls in Finland, 2002-2012. Papillomavirus Res 2017; 3: 91-6.

78. Auger N, Quach C, Healy-Profitós J, Dinh T, Chassé M. Early predictors of Guillain-Barré syndrome in the life course of women. International Journal of Epidemiology 2018; 47(1): 280-8.

79. Hafsteinsdóttir B, Ólafsson E, Jakobsson F. Incidence and outcome of Guillain-Barré syndrome in Iceland: A population-based study. Acta Neurologica Scandinavica 2018; 138(5): 454-8.

80. Matsui N, Nodera H, Kuzume D, et al. Guillain-Barré syndrome in a local area in Japan, 2006-2015: an epidemiological and clinical study of 108 patients. Eur J Neurol 2018; 25(5): 718-24.

81. Abdel-Fattah A, El-Gilany AH, El-Masry R, Kanddeel A. Acute flaccid paralysis in North East Delta, Egypt: A retrospective analysis of prospectively collected surveillance data. Journal of Infection and Public Health 2019; 12(5): 714-9.

82. Al-Hakem H, Sindrup SH, Andersen H, et al. Guillain–Barré syndrome in Denmark: a population-based study on epidemiology, diagnosis and clinical severity. Journal of Neurology 2019; 266(2): 440-9.

83. Benedetti L, Briani C, Beronio A, et al. Increased incidence of axonal Guillain-Barré syndrome in La Spezia area of Italy: A 13-year follow-up study. Journal of the Peripheral Nervous System 2019; 24(1): 80-6.

84. Granieri E, Andreasi NG, De Martin P, et al. Incidence study of Guillain-Barré syndrome in the province of Ferrara, Northern Italy, between 2003 and 2017. A 40-year follow-up. Neurological Sciences 2019; 40(3): 603-9.

85. Koul R, Al-Fuitaisi A, Macki N, et al. Incidence of Guillain-Barre's Syndrome in Children under 15 Years of Age in Oman. Journal of Pediatric Neurology 2019; 17(6): 206-9.

86. Levison LS, Thomsen RW, Christensen DH, Mellemkjær T, Sindrup SH, Andersen H. Guillain-barré syndrome in Denmark: Validation of diagnostic codes and a population-based nationwide study of the incidence in a 30-year period. Clinical Epidemiology 2019; 11: 275-83.

87. Momen AA, Shakurnia A, Momen M. Eleven-year surveillance of acute flaccid paralysis in Southwestern Iran. Turkish Journal of Pediatrics 2019; 61(4): 544-51.

88. Lee H, Kang HY, Jung SY, Lee YM. Incidence of guillain-barré syndrome is not associated with influenza vaccination in the elderly. Vaccines 2020; 8(3): 1-13.

89. Leung J, Sejvar JJ, Soares J, Lanzieri TM. Guillain-Barré syndrome and antecedent cytomegalovirus infection, USA 2009-2015. Neurol Sci 2020; 41(4): 885-91.

90. Levison LS, Thomsen RW, Markvardsen LK, Christensen DH, Sindrup SH, Andersen H. Pediatric Guillain-Barré Syndrome in a 30-Year Nationwide Cohort. Pediatric Neurology 2020; 107: 57-63.

91. Malta JMAS, Ramalho WM. Increase in Guillain-Barré syndrome hospitalizations in Brazil: an ecological study. Epidemiologia e servicos de saude : revista do Sistema Unico de Saude do Brasil 2020; 29(4): e2020056.

92. Stojanov A, Berisavac I, Bozovic I, et al. Incidence and mortality rates of Guillain-Barré syndrome in Serbia. Journal of the Peripheral Nervous System 2020; 25(4): 350-5.

93. Carey IM, Banchoff E, Nirmalananthan N, et al. Prevalence and incidence of neuromuscular conditions in the UK between 2000 and 2019: A retrospective study using primary care data. PLoS ONE 2021; 16(12 December).

94. Chiesa M, Decima R, Bertinat A, et al. Incidence of Guillain-Barré syndrome in an Uruguayan population. A prospective cohort study. Journal of the Peripheral Nervous System 2021; 26(2): 209-15.

95. Filosto M, Cotti Piccinelli S, Gazzina S, et al. Guillain-Barre&acute; Syndrome and COVID-19: An observational multicentre study from two Italian hotspot regions. Journal of Neurology, Neurosurgery and Psychiatry 2021; 92(7): 751-6.

96. Fragiel M, Miró Ò, Llorens P, et al. Incidence, clinical, risk factors and outcomes of Guillain-Barré in Covid-19. Annals of Neurology 2021; 89(3): 598-603.

97. Kasemsap N, Vorasoot N, Kongbunkiat K, et al. The epidemiology of Guillain-Barré syndrome in Thailand over 13 years (2005-2017): A nationwide population-based retrospective cohort study. J Peripher Nerv Syst 2021; 26(2): 202-8.

98. Kim AY, Lee H, Lee YM, Kang HY. Epidemiological features and economic burden of guillain-barré syndrome in south korea: A nationwide population-based study. Journal of Clinical Neurology (Korea) 2021; 17(2): 257-64.

99. Tonekaboni SH, Mahmoudi S, Abdollahgorji F, et al. Epidemiology of Guillain-Barré Syndrome in Iranian Children Aged 0-15 Years (2008-2013). Iranian Journal of Child Neurology 2021; 15(4): 27-34.

100. Wachira VK, Nascimento GL, Peixoto HM, de Oliveira MRF. Burden of Disease of Guillain–Barré Syndrome in Brazil before and during the Zika virus epidemic 2014–2016. Tropical Medicine and International Health 2021; 26(1): 66-81.

101. Yoon HH, Park JY, Kim SY, et al. Epidemiology of Demyelinating Diseases in Korean Pediatric Patients. Journal of Child Neurology 2021; 36(2): 141-7.

102. Hosseininezhad M, Khatami SS, Saadat S, et al. Ten years evaluation of epidemiology- and mortality-related factors in adults and children with Guillain-Barré syndrome in the north of Iran. Neurological Sciences 2022; 43(3): 1929-38.

103. Nasreen S, Calzavara A, Buchan SA, et al. Background incidence rates of adverse events of special interest related to COVID-19 vaccines in Ontario, Canada, 2015 to 2020, to inform COVID-19 vaccine safety surveillance. Vaccine 2022; 40(24): 3305-12.

104. Yi SW, Lee JH, Hong JM, Choi YC, Park HJ. Incidence, Disability, and Mortality in Patients With Guillain-Barré Syndrome in Korea: A Nationwide Population-Based Study. Journal of Clinical Neurology (Korea) 2022; 18(1): 48-58.

105. Zheng P, Tian DC, Xiu Y, Wang Y, Shi FD. Incidence of Guillain-Barré syndrome (GBS) in China: A national population-based study. Lancet Reg Health West Pac 2022; 18: 100302.

106. Kinnunen E, Junttila O, Haukka J, Hovi T. Nationwide oral poliovirus vaccination campaign and the incidence of Guillain-Barré Syndrome. Am J Epidemiol 1998; 147(1):69-73.

107. Govoni V, Granieri E, Manconi M, Capone J, Casetta I. Is there a decrease in Guillain-Barré syndrome incidence after bovine ganglioside withdrawal in Italy? A population-based study in the Local Health District of Ferrara, Italy. J Neurol Sci 2003; 216(1):99-103.

108. Hui AC, Chow KM, Tang AS, Fu M, Kay R, Wong KS. Electrophysiological, clinical and epidemiological study of Guillain-Barre Syndrome in Hong Kong Chinese. J Clin Neurosci 2005; 12(2):134-136.

109. Rocha MS, Brucki SM, Carvalho AA, Lima UW. Epidemiologic features of Guillain-Barré syndrome in São Paulo, Brazil. Arq Neuropsiquiatr 2004; 62(1):33-37.

110. Arami MA, Yazdchi M, Khandaghi R. Epidemiology and characteristics of Guillain-Barré syndrome in the northwest of Iran. Ann Saudi Med 2006; 26(1):22-27.

111. Howlett WP, Vedeler CA, Nyland H, Aarli JA. Guillain-Barré syndrome in northern Tanzania: a comparison of epidemiological and clinical findings with western Norway. Acta Neurol Scand 1996; 93(1):44-49.

112. Hughes RA, Charlton J, Latinovic R, Gulliford MC. No association between immunization and Guillain-Barré syndrome in the United Kingdom, 1992 to 2000. Arch Intern Med 2006; 166(12):1301-1304.

113. MacDonald BK, Cockerell OC, Sander JW, Shorvon SD. The incidence and lifetime prevalence of neurological disorders in a prospective community-based study in the UK. Brain 2000; 123 ( Pt 4):665-676.

114. McLean M, Duclos P, Jacob P, Humphreys P. Incidence of Guillain-Barré syndrome in Ontario and Quebec, 1983-1989, using hospital service databases. Epidemiology 1994; 5(4):443-448.

115. Lehmann HC, Köhne A, Meyer zu Hörste G, Kieseier BC. Incidence of Guillain-Barré syndrome in Germany. J Peripher Nerv Syst 2007; 12(4):285.

116. Molinero MR, Varon D, Holden KR, Sladky JT, Molina IB, Cleaves F. Epidemiology of childhood Guillain-Barré syndrome as a cause of acute flaccid paralysis in Honduras: 1989-1999. J Child Neurol 2003; 18(11):741-747.

117. Hart DE, Rojas LA, Rosário JA, Recalde H, Román GC. Childhood Guillain-Barré syndrome in Paraguay, 1990 to 1991. Ann Neurol 1994; 36(6):859-863.

118. Beghi E, Kurland LT, Mulder DW, Wiederholt WC. Guillain-Barré syndrome. Clinicoepidemiologic features and effect of influenza vaccine. Arch Neurol 1985; 42(11):1053-1057.

119. Ismail EA, Shabani IS, Badawi M, et al. An epidemiologic, clinical, and therapeutic study of childhood Guillain-Barré syndrome in Kuwait: is it related to the oral polio vaccine? J Child Neurol 1998; 13(10):488-492.

120. Rantala H, Cherry JD, Shields WD, Uhari M. Epidemiology of Guillain-Barré syndrome in children: relationship of oral polio vaccine administration to occurrence. J Pediatr 1994; 124(2):220-223.

121. Jiang GX, Cheng Q, Link H, de Pedro-Cuesta J. Epidemiological features of Guillain-Barré syndrome in Sweden, 1978-93. J Neurol Neurosurg Psychiatry 1997; 62(5):447-453.

122. Schonberger LB, Bregman DJ, Sullivan-Bolyai JZ, et al. Guillain-Barre syndrome following vaccination in the National Influenza Immunization Program, United States, 1976--1977. Am J Epidemiol 1979; 110(2):105-123.

# Supplementary file 8. Quality scores of included studies

| publication year | author | Q1 | Q2 | Q3 | Q4 | Q5 | Q6 | Q7 | Q8 | Score |
| --- | --- | --- | --- | --- | --- | --- | --- | --- | --- | --- |
| 1976 | Andersen, O. | Yes | Yes | Not Applicable | Not Applicable | Yes | Yes | Yes | No | 0.625 |
| 1979 | Hogg, J. E. | Yes | Yes | Not Applicable | Not Applicable | Yes | Yes | Yes | Yes | 0.75 |
| 1982 | Haberman, S. | Yes | Yes | Not Applicable | Not Applicable | Yes | Yes | Yes | Yes | 0.75 |
| 1982 | Kaplan, J. E. | Yes | Yes | Not Applicable | Not Applicable | Yes | Yes | No | No | 0.5 |
| 1985 | Bak, P. | Yes | Yes | Not Applicable | Not Applicable | Yes | Yes | Yes | Unclear | 0.625 |
| 1985 | Larsen, J. P. | Yes | Yes | Not Applicable | Not Applicable | Yes | Yes | Yes | No | 0.625 |
| 1987 | Hankey, G. J. | Yes | Yes | Not Applicable | Not Applicable | Yes | Yes | Yes | Unclear | 0.625 |
| 1987 | Radhakrishnan, K. | Yes | Yes | Not Applicable | Not Applicable | Yes | Yes | Yes | No | 0.625 |
| 1988 | Hallas, J. | Yes | Yes | Not Applicable | Not Applicable | Yes | Yes | Yes | No | 0.625 |
| 1989 | Storey, E. | Unclear | Yes | Not Applicable | Not Applicable | Yes | Yes | Yes | Unclear | 0.5 |
| 1990 | Winner, S. J. | Yes | Yes | Not Applicable | Not Applicable | Yes | Yes | Yes | Yes | 0.75 |
| 1991 | Farkkila, M. | Yes | Yes | Not Applicable | Not Applicable | Yes | Unclear | Yes | No | 0.5 |
| 1991 | Koobatian, T. J. | Yes | Yes | Not Applicable | Not Applicable | Yes | Yes | Yes | No | 0.625 |
| 1991 | Paolino, E. | Yes | Yes | Not Applicable | Not Applicable | Yes | Yes | Yes | Yes | 0.75 |
| 1991 | Rantala, H. | Yes | Yes | Not Applicable | Not Applicable | Yes | Yes | Yes | No | 0.625 |
| 1993 | Matias-Guiu, J. | Yes | Unclear | Not Applicable | Not Applicable | Yes | Yes | Yes | Yes | 0.625 |
| 1993 | Sridharan, G. V. | Yes | Yes | Not Applicable | Not Applicable | Yes | Yes | Yes | Yes | 0.75 |
| 1994 | Hung, K. L. | Yes | Yes | Not Applicable | Not Applicable | Yes | Yes | Yes | No | 0.625 |
| 1994 | Sedano, M. J. | Yes | Yes | Not Applicable | Not Applicable | Yes | Yes | Yes | Yes | 0.75 |
| 1995 | Jiang, G. X. | Yes | Yes | Not Applicable | Not Applicable | Yes | Yes | Yes | Yes | 0.75 |
| 1995 | Kusumi, M. | Yes | Yes | Not Applicable | Not Applicable | Yes | Yes | Unclear | No | 0.5 |
| 1996 | Beghi, E. | Yes | Yes | Not Applicable | Not Applicable | Yes | Yes | Yes | Yes | 0.75 |
| 1996 | Govoni, V. | Yes | Yes | Not Applicable | Not Applicable | Yes | Yes | Yes | Yes | 0.75 |
| 1997 | Emilia-Romagna Study Group on Clinical and Epidemiological Problems in Neurology | Yes | Yes | Not Applicable | Not Applicable | Yes | Yes | Yes | Yes | 0.75 |
| 1997 | Jiang, G. X. | Yes | Yes | Not Applicable | Not Applicable | Yes | Yes | Yes | No | 0.625 |
| 1997 | Olivé, J. M. | Yes | Yes | Not Applicable | Not Applicable | Yes | Yes | Yes | No | 0.625 |
| 1997 | Prevots, D. R. | Yes | No | Not Applicable | Not Applicable | Yes | Yes | No | No | 0.375 |
| 1998 | Casmiro, M. | Yes | Yes | Not Applicable | Not Applicable | Yes | Yes | Yes | No | 0.625 |
| 1998 | Rees, J. H. | Yes | Yes | Not Applicable | Not Applicable | Yes | Yes | Yes | Yes | 0.75 |
| 1999 | Govoni, V. | Yes | Yes | Not Applicable | Not Applicable | Yes | Yes | Yes | Yes | 0.75 |
| 2000 | Cheng, Q. | Yes | Yes | Not Applicable | Not Applicable | Yes | Yes | Yes | No | 0.625 |
| 2000 | Hong, X. | Unclear | Unclear | 3 | 3 | Unclear | Unclear | Unclear | Unclear | 0 |
| 2000 | Van Koningsveld, R. | Yes | Yes | Not Applicable | Not Applicable | Yes | Yes | Yes | No | 0.625 |
| 2001 | Cuadrado, J. I. | Yes | Yes | Not Applicable | Not Applicable | Yes | Yes | Yes | Yes | 0.75 |
| 2001 | van Koningsveld, R. | Yes | Yes | Not Applicable | Not Applicable | Yes | Yes | Yes | Yes | 0.75 |
| 2002 | Cheng, Q. | Yes | Yes | Not Applicable | Not Applicable | Yes | Yes | Yes | Yes | 0.75 |
| 2002 | Dias-Tosta, E. | Yes | Yes | Not Applicable | Not Applicable | Yes | Yes | Yes | No | 0.625 |
| 2002 | Varsik, P. | Yes | Yes | Not Applicable | Not Applicable | Yes | Yes | Yes | No | 0.625 |
| 2003 | Chiò, A. | Yes | Yes | Not Applicable | Not Applicable | Yes | Yes | Yes | Yes | 0.75 |
| 2003 | Morris, A. M. S. | Yes | Yes | Not Applicable | Not Applicable | Yes | Yes | Yes | No | 0.625 |
| 2003 | Potter, R. C. | Yes | Yes | Not Applicable | Not Applicable | Yes | Yes | No | Yes | 0.625 |
| 2004 | Bogliun, G. | Yes | Yes | Not Applicable | Not Applicable | Yes | Yes | Yes | Yes | 0.75 |
| 2004 | Chroni, E. | Yes | Yes | Not Applicable | Not Applicable | Yes | Yes | Yes | Yes | 0.75 |
| 2004 | Cuadrado, J. I. | Yes | Yes | Not Applicable | Not Applicable | Yes | Yes | Yes | No | 0.625 |
| 2004 | Hussain, I. H. M. I. | Yes | Yes | Not Applicable | Not Applicable | Yes | Yes | Yes | No | 0.625 |
| 2004 | Zhang, Y. | Yes | Yes | Not Applicable | Not Applicable | Yes | Yes | Yes | No | 0.625 |
| 2007 | Barzegar, M. | Yes | Yes | Not Applicable | Not Applicable | Yes | Yes | Yes | Yes | 0.75 |
| 2007 | Eaton, W. W. | Yes | Yes | Not Applicable | Not Applicable | Yes | Yes | Unclear | Yes | 0.625 |
| 2007 | Howitz, M. F. | Yes | Yes | Not Applicable | Not Applicable | Yes | Yes | Unclear | No | 0.5 |
| 2007 | Markoula, S. | Yes | Yes | Not Applicable | Not Applicable | Yes | Yes | Yes | No | 0.625 |
| 2008 | Alshekhlee, A. | Yes | Yes | Yes | Yes | Yes | Yes | Yes | No | 0.875 |
| 2008 | Deceuninck, G. | Yes | Yes | Not Applicable | Not Applicable | Yes | Yes | Yes | Yes | 0.75 |
| 2008 | Hauck, L. J. | Yes | Yes | Not Applicable | Not Applicable | Yes | Yes | Yes | No | 0.625 |
| 2008 | Koul, R. | Yes | Yes | Not Applicable | Not Applicable | Yes | Yes | Yes | No | 0.625 |
| 2010 | Kang, J. H. | Yes | Yes | Not Applicable | Not Applicable | Yes | Yes | Yes | No | 0.625 |
| 2011 | Ruzante, J. M. | Yes | Yes | Yes | Yes | Yes | Yes | Yes | No | 0.875 |
| 2011 | van der Maas, N. A. | Yes | Yes | Yes | Yes | Yes | Yes | Yes | Yes | 1 |
| 2012 | Kandil, M. R. | Yes | Yes | Yes | No | Yes | Yes | Yes | No | 0.75 |
| 2012 | Lee, C. D. | Yes | Yes | Not Applicable | Not Applicable | Yes | Yes | Yes | No | 0.625 |
| 2012 | Shui, I. M. | Yes | Yes | Not Applicable | Not Applicable | Yes | Yes | Yes | Yes | 0.75 |
| 2013 | Jasem, J. | Yes | Yes | Not Applicable | Not Applicable | Yes | Yes | Yes | Yes | 0.75 |
| 2014 | Chen, Y. | Yes | Yes | Not Applicable | Not Applicable | Yes | Yes | Yes | No | 0.625 |
| 2014 | Hense, S. | Yes | Yes | Not Applicable | Not Applicable | Yes | Yes | Yes | No | 0.625 |
| 2014 | Peric, S. | Yes | Yes | Not Applicable | Not Applicable | Yes | Yes | Yes | No | 0.625 |
| 2015 | Benedetti, M. D. | Yes | Yes | Yes | No | Yes | Yes | Yes | Yes | 0.875 |
| 2015 | Huang, W. C. | Yes | Yes | Not Applicable | Not Applicable | Yes | Yes | Yes | Yes | 0.75 |
| 2015 | Sipila, J. O. T. | Yes | Yes | Not Applicable | Not Applicable | Yes | Yes | Yes | No | 0.625 |
| 2016 | Liou, L. S. | Yes | Yes | Not Applicable | Not Applicable | Yes | Yes | Yes | Yes | 0.75 |
| 2016 | Momen, A. A. | Yes | Yes | Not Applicable | Not Applicable | Yes | Yes | Yes | No | 0.625 |
| 2016 | Rivera-Lillo, G. | Yes | Yes | Not Applicable | Not Applicable | Yes | Yes | Yes | No | 0.625 |
| 2016 | Suryapranata, F. S. T. | Yes | Yes | Not Applicable | Not Applicable | Yes | Yes | Yes | No | 0.625 |
| 2017 | Delannoy, A. | Yes | Yes | Not Applicable | Not Applicable | Yes | Yes | Yes | Yes | 0.75 |
| 2017 | Momen, A. A. | Yes | Yes | Not Applicable | Not Applicable | Yes | Yes | Yes | Yes | 0.75 |
| 2017 | Pastula, D. M. | Yes | Yes | Not Applicable | Not Applicable | Yes | Yes | Yes | No | 0.625 |
| 2017 | Salinas, J. L. | Yes | Yes | Not Applicable | Not Applicable | Yes | Yes | Yes | No | 0.625 |
| 2017 | Sipila, J. O. T. | Yes | Yes | Not Applicable | Not Applicable | Yes | Yes | Yes | No | 0.625 |
| 2017 | Skufca, J. | Yes | Yes | Not Applicable | Not Applicable | Yes | Yes | Yes | Yes | 0.75 |
| 2018 | Auger, N. | Yes | Yes | Not Applicable | Not Applicable | Yes | Yes | Yes | Yes | 0.75 |
| 2018 | Hafsteinsdóttir, B. | Yes | Yes | Not Applicable | Not Applicable | Yes | Yes | Yes | No | 0.625 |
| 2018 | Matsui, N. | Yes | Yes | Not Applicable | Not Applicable | Yes | Yes | Yes | Yes | 0.75 |
| 2019 | Abdel-Fattah, A. | Yes | Yes | Not Applicable | Not Applicable | Yes | Yes | Yes | No | 0.625 |
| 2019 | Al-Hakem, H. | Yes | Yes | Not Applicable | Not Applicable | Yes | Yes | Yes | Yes | 0.75 |
| 2019 | Benedetti, L. | Yes | Yes | Not Applicable | Not Applicable | Yes | Yes | Yes | No | 0.625 |
| 2019 | Granieri, E. | Yes | Yes | Not Applicable | Not Applicable | Yes | Yes | Yes | Yes | 0.75 |
| 2019 | Koul, R. | Yes | Yes | Not Applicable | Not Applicable | Yes | Yes | Yes | No | 0.625 |
| 2019 | Levison, L. S. | Yes | Yes | Not Applicable | Not Applicable | Yes | Yes | Yes | Yes | 0.75 |
| 2019 | Momen, A. A. | Yes | Yes | Not Applicable | Not Applicable | Yes | Yes | Yes | No | 0.625 |
| 2020 | Lee, H. | Yes | Yes | Not Applicable | Not Applicable | Yes | Yes | Yes | No | 0.625 |
| 2020 | Leung, J. | Yes | Yes | Not Applicable | Not Applicable | Yes | Yes | Yes | No | 0.625 |
| 2020 | Levison, L. S. | Yes | Yes | Not Applicable | Not Applicable | Yes | Yes | Yes | Yes | 0.75 |
| 2020 | Malta, J. M. A. S. | Yes | Yes | Not Applicable | Not Applicable | Yes | Yes | Yes | No | 0.625 |
| 2020 | Stojanov, A. | Yes | Yes | Not Applicable | Not Applicable | Yes | No | Yes | Yes | 0.625 |
| 2021 | Carey, I. M. | Yes | Yes | Not Applicable | Not Applicable | Yes | Yes | Yes | Yes | 0.75 |
| 2021 | Chiesa, M. | Yes | Yes | Not Applicable | Not Applicable | Yes | Yes | Yes | Yes | 0.75 |
| 2021 | Filosto, M. | Yes | Yes | Not Applicable | Not Applicable | Yes | Yes | Yes | No | 0.625 |
| 2021 | Fragiel, M. | Yes | Yes | Not Applicable | Not Applicable | Yes | Yes | Yes | No | 0.625 |
| 2021 | Kasemsap, N. | Yes | Yes | Not Applicable | Not Applicable | Yes | Yes | Yes | No | 0.625 |
| 2021 | Kim, A. Y. | Yes | Yes | Not Applicable | Not Applicable | Yes | Yes | Yes | Yes | 0.75 |
| 2021 | Tonekaboni, S. H. | Yes | Yes | Not Applicable | Not Applicable | Yes | Yes | Yes | No | 0.625 |
| 2021 | Wachira, V. K. | Yes | Yes | Not Applicable | Not Applicable | Yes | Yes | No | No | 0.5 |
| 2021 | Yoon, H. H. | Yes | Yes | Not Applicable | Not Applicable | Yes | Yes | Yes | No | 0.625 |
| 2022 | Hosseininezhad, M. | Yes | Yes | Not Applicable | Not Applicable | Yes | Yes | Yes | No | 0.625 |
| 2022 | Nasreen, S. | Yes | Yes | Not Applicable | Not Applicable | Yes | Yes | Yes | Yes | 0.75 |
| 2022 | Yi, S. W. | Yes | Yes | Not Applicable | Not Applicable | Yes | Yes | Yes | Yes | 0.75 |
| 2022 | Zheng, P. | Yes | Yes | Not Applicable | Not Applicable | Yes | Yes | Yes | Yes | 0.75 |
| 1998 | Kinnunen, E. | Yes | Yes | Not Applicable | Not Applicable | Yes | Yes | Yes | Yes | 0.75 |
| 2003 | Govoni, V. | Yes | Yes | Not Applicable | Not Applicable | Yes | Yes | Yes | Yes | 0.75 |
| 2005 | Hui, A. C. | Yes | Yes | Not Applicable | Not Applicable | Yes | Yes | Yes | 0 | 0.625 |
| 2004 | Rocha, M. S. | Yes | Yes | Not Applicable | Not Applicable | Yes | Yes | Yes | 0 | 0.625 |
| 2006 | Arami, M. A. | Yes | Yes | Not Applicable | Not Applicable | Yes | Yes | Yes | 0 | 0.625 |
| 1996 | Howlett, W. P. | Yes | Yes | Not Applicable | Not Applicable | Yes | Yes | Yes | 0 | 0.625 |
| 2006 | Hughes, R. A. | Yes | Yes | Not Applicable | Not Applicable | Yes | Yes | Yes | Yes | 0.75 |
| 2000 | MacDonald, B. K. | Yes | Yes | Not Applicable | Not Applicable | Yes | Yes | Yes | 0 | 0.625 |
| 1994 | McLean, M. | Yes | Yes | Not Applicable | Not Applicable | Yes | Yes | Yes | Yes | 0.75 |
| 2007 | Lehmann, H. C. | Yes | Yes | Not Applicable | Not Applicable | Yes | Yes | 0 | 0 | 0.5 |
| 2003 | Molinero, M. R. | Yes | Yes | Not Applicable | Not Applicable | Yes | Yes | Yes | 0 | 0.625 |
| 1994 | Hart, D. E. | Yes | Yes | Not Applicable | Not Applicable | Yes | Yes | Yes | 0 | 0.625 |
| 1985 | Beghi, E. | Yes | Yes | Not Applicable | Not Applicable | Yes | Yes | Yes | Yes | 0.75 |
| 1998 | Ismail, E. A. | Yes | Yes | Not Applicable | Not Applicable | Yes | Yes | Yes | Yes | 0.75 |
| 1994 | Rantala, H. | Yes | Yes | Not Applicable | Not Applicable | Yes | Yes | Yes | Yes | 0.75 |
| 1997 | Jiang, G. X. | Yes | Yes | Not Applicable | Not Applicable | Yes | Yes | Yes | 0 | 0.625 |
| 1979 | Schonberger, L. B. | Yes | Yes | Not Applicable | Not Applicable | Yes | Yes | Yes | 0 | 0.625 |

# Supplementary file 9. Results of meta regression

| **Study level covariates** | ***P*-value** |
| --- | --- |
| Subcontinent |  |
| North Europe | Ref |
| Australia and New Zealand | 0.033 |
| East Asia | 0.066 |
| East Europe | 0.652 |
| North Africa | 0.213 |
| North America | 0.823 |
| South America | 0.081 |
| South Asia | 0.646 |
| South Europe | 0.116 |
| Southeast Asia | 0.031 |
| West Asia | 0.146 |
| West Europe | 0.165 |
| Study period | 0.022 |
| Study quality | 0.070 |
| Economic level |  |
| Low-income | Ref |
| Lower-middle-income | 0.399 |
| Upper-middle-income | 0.866 |
| High-income | 0.334 |
| Average age | 0.160 |
| Male-to-Female ratio | 0.092 |

# Supplementary file 10. Funnel plot and Egger’s test


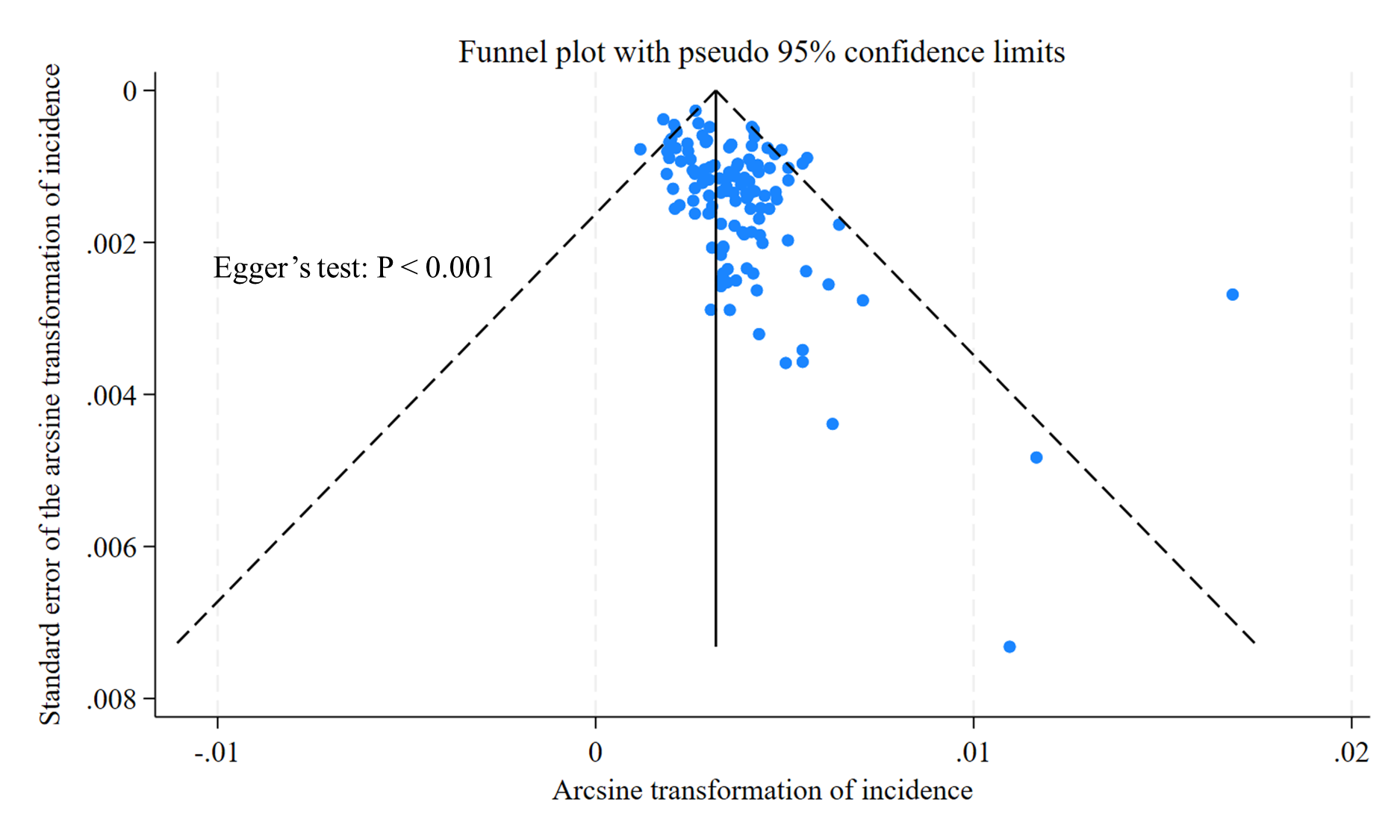


# Supplementary file 11. Linear regression between different factors with national GBS incidence

| **Factors** | **Coefficient** | ***P*-value** |
| --- | --- | --- |
| Socio-demographic index | -0.2300148 | 0.840 |
| Physician density | -0.0076673 | 0.513 |
| Human development index | -0.2959876 | 0.853 |
| Proportion of the elderly population | 0.0020319 | 0.956 |
| Incidence of enteric infections | 0.0000185 | 0.007 |
| Incidence of respiratory infections | 4.78e-06 | 0.214 |
